# Fog Task Scheduling Using Quality-Source-Driven Multi-Anchor Synchronized Search Algorithm

**DOI:** 10.3390/biomimetics11060392

**Published:** 2026-06-03

**Authors:** Haitao Xie, Zhuo Luo, Zhiwei Ye, Wen Zhou, Xianjing Zhou, Donglei Xu, Mingming Zhao

**Affiliations:** 1 School of Computer Science and Artificial Intelligence, Hubei University of Technology, Wuhan 430068, China; 19991035@hbut.edu.cn (H.X.); 102401148@hbut.edu.cn (Z.L.); zw_mmwh@whu.edu.cn (W.Z.); 2 Hubei Provincial Key Laboratory of Green Intelligent Computing Power Network, Hubei University of Technology, Wuhan 430068, China; 3 Wuhan Zhuoer Information Technology Co., Ltd., Wuhan 430312, China; zxjzallcorp@163.com; 4 Wuhan Fiberhome Technical Services Co., Ltd., Wuhan 430205, China; dlxu@fiberhome.com (D.X.); mmzhao@fiberhome.com (M.Z.)

**Keywords:** IoT–Fog task scheduling, quality-source anchors, multi-anchor synchronization, metaheuristic algorithm, large-scale scheduling

## Abstract

Efficient task scheduling in heterogeneous IoT–Fog environments is challenging due to limited fog resources, diverse task demands, and conflicting QoS objectives. This paper proposes ASQS, a Quality-Source-driven Multi-Anchor Synchronized Search algorithm for IoT–Fog task scheduling. ASQS is biomimetically motivated by collective search behaviors in natural systems, where distributed exploration, collective memory, and probabilistic cooperation support an exploration–exploitation balance. Specifically, ASQS constructs quality layers from candidate schedules, extracts representative quality-source anchors, and reuses them through an ACO-inspired probabilistic synchronization mechanism, thereby improving the utilization of high-quality historical search information. FNO-based search and Lévy-flight perturbation are further incorporated to enhance directional guidance and long-range exploration. Experiments on 33 benchmark functions, ablation studies, sensitivity analysis, standard fog scheduling scenarios, and large-scale task-intensive scenarios were conducted to evaluate ASQS. The results show that ASQS achieves competitive optimization accuracy, stable convergence, and superior comprehensive scheduling performance in terms of fitness, makespan, latency, load balance, and constraint handling. In particular, the large-scale experiment with 100 fog nodes and up to 8000 IoT tasks verifies the scalability of ASQS under heavy workload pressure. Statistical tests further confirm the reliability of the observed improvements. These results demonstrate that ASQS is an effective, scalable, and biomimetically motivated optimizer for IoT–Fog task scheduling.

## 1. Introduction

**Figure 1:**
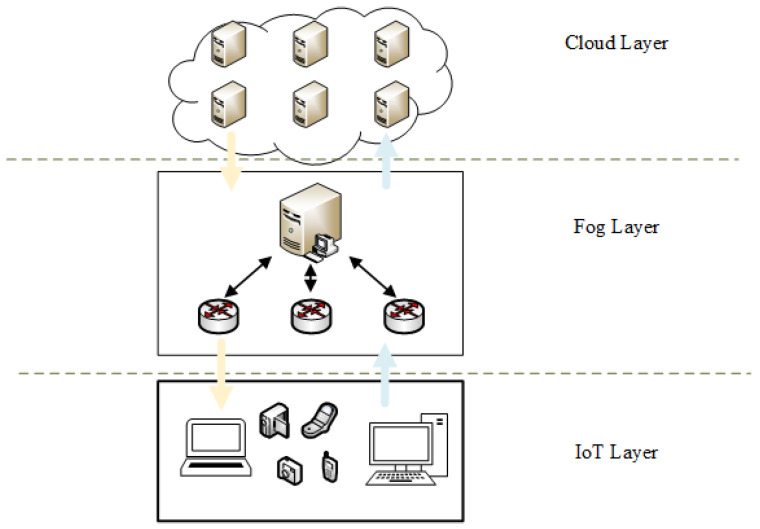
Typical fog computing architecture consisting of the IoT layer, fog layer, and cloud layer, where the arrows indicate the direction of data flow and communication between different layers.

The rapid proliferation of IoT applications has generated a large number of latency-sensitive task requests from edge devices. Sole reliance on remote cloud servers is often inefficient, as long-distance data transmission may introduce substantial latency and bandwidth pressure [1,2]. Fog computing alleviates this limitation by deploying computing resources closer to IoT devices; however, the constrained and heterogeneous nature of fog resources makes task scheduling a critical challenge. Inefficient scheduling may result in node overload, prolonged completion time, and degraded quality of service, highlighting the necessity of efficient IoT–Fog task scheduling in latency-sensitive environments [3,4]. Figure 1 illustrates a typical three-layer fog computing architecture, where IoT devices generate task requests, fog nodes provide nearby computing resources, and cloud servers offer centralized support for large-scale processing.

Fog task scheduling is challenging because both resources and workloads are highly heterogeneous. Fog nodes, including gateways, routers, base stations, and edge servers, usually differ in computing capacity, memory, bandwidth, energy consumption, and availability, while IoT tasks have diverse requirements in CPU cycles, input data size, memory demand, priority, and deadlines. The scheduler must assign each task to a suitable fog node under resource and deadline constraints, forming a discrete task–node assignment problem. Moreover, multiple QoS objectives, such as makespan, response latency, energy consumption, load balance, resource utilization, and task completion rate, are often conflicting and must be optimized simultaneously [5,6]. Existing time-aware and multi-objective scheduling studies have attempted to improve deadline satisfaction and global scheduling quality [5]; however, reliability, dependability, and dynamic decision-making remain important concerns in practical IoT–Fog systems [7,8,9]. Therefore, fog task scheduling is generally treated as an NP-hard combinatorial optimization problem.

Recent studies have increasingly moved beyond conventional heuristic and metaheuristic schedulers toward hybrid, learning-based, and adaptive optimization frameworks for IoT–Fog task scheduling, including deep reinforcement learning and fuzzy deep reinforcement learning-based scheduling methods [10,11]. For example, Heirati et al. integrated deep learning, reinforcement learning, and metaheuristic optimization to improve energy efficiency, task completion time, and QoS [12]. Yu et al. developed NF-MORL, which combines Takagi–Sugeno fuzzy inference with multi-objective actor–critic learning to optimize completion time, energy consumption, cost, and fault tolerance under uncertainty [13]. Mindil et al. proposed QBDS, a quantum-inspired scheduler that uses priority-aware ranking, sinusoidal biasing, and penalty-aware multi-objective evaluation to improve makespan, energy, cost, load balancing, response time, and scalability [1].

Despite extensive research on IoT–Fog scheduling, several gaps remain unresolved. First, heterogeneous tasks, resource-constrained fog nodes, conflicting QoS objectives, and dynamic system states still make task-node assignment a difficult optimization problem [14,15,16,17]. Second, although heuristic, metaheuristic, and learning-based schedulers have achieved improvements, population-based search remains sensitive to parameter settings and is still prone to premature convergence and exploration–exploitation imbalance [12,13,18,19]. Third, the main weakness of many hybrid metaheuristic schedulers is not the absence of search operators but the absence of a structured mechanism for quality-source management: high-quality search experience is often generated but not explicitly extracted, archived, and reused as multi-source guidance across promising regions [12,18,19]. Fourth, many studies still under-specify objective aggregation, penalty-based constraint handling, parameter sensitivity, scalability behavior, and fair comparison protocols, thereby limiting reproducibility and the reliability of performance claims [16,17,20,21].

Motivated by these observations, this paper proposes ASQS, a quality-source-driven multi-anchor synchronized search algorithm for resource-constrained IoT–Fog task scheduling. The design of ASQS is closely related to biomimetic optimization principles observed in natural collective systems, such as ant colonies, bird flocks, and animal foraging groups, where distributed exploration, collective memory, and probabilistic cooperation are commonly used to balance exploration and exploitation. Unlike conventional hybrid metaheuristics that mainly accumulate multiple operators or rely heavily on a single global-best solution, ASQS shifts the focus to quality-source management. Specifically, high-quality search experience is extracted from layered populations, archived as reusable anchors, and probabilistically reused through ACO-inspired selection and anchor-seeding to provide multi-source guidance over multiple promising regions. Combined with FNO-based directional search and Lévy-flight perturbation, which supports occasional long-range exploration consistent with biological foraging behavior, the proposed framework alleviates excessive single-elite dependence and forms a biomimetically motivated search process for IoT–Fog scheduling.

The main contributions of this paper are summarized as follows:

1. Establishment of a resource-constrained IoT–Fog scheduling model with explicit task-node encoding, QoS-aware objective aggregation, and penalty-based treatment of resource and deadline violations.
2. Introduction of a biomimetically motivated quality-source management mechanism that extracts high-quality solutions from layered populations and organizes them as reusable anchors, reflecting collective memory and multi-source experience reuse in natural systems.
3. Design of an ACO-inspired multi-anchor synchronized search strategy that probabilistically reuses archived anchors as multi-source guidance, mimicking indirect information sharing and cooperative path reinforcement while mitigating single-elite dependence.
4. Comprehensive validation based on benchmark-function tests, IoT–Fog scheduling simulations, algorithm comparisons, ablation studies, sensitivity analysis, scalability evaluation, and statistical tests.

The remainder of this paper is organized as follows: Section 2 reviews related work on IoT–Fog task scheduling, metaheuristic scheduling methods, and search experience reuse. Section 3 introduces the component roles and design motivation of the proposed ASQS. Section 4 presents the ASQS algorithm in detail, including solution encoding, fitness evaluation, search operators, quality-source archive construction, and the overall procedure. Section 5 reports the experimental studies, including benchmark-function comparisons, sensitivity analysis, ablation experiments, and IoT–Fog task scheduling experiments. Finally, Section 6 concludes this paper and discusses future research directions.

## 2. Related Works

### 2.1. IoT–Fog Task Scheduling Models and QoS Objectives

IoT–Fog task scheduling has been widely studied as a resource-constrained task-node assignment problem, where heterogeneous IoT tasks are allocated to heterogeneous fog nodes under capacity, latency, energy, and deadline constraints [22,23,24,25]. Existing studies have considered various QoS objectives, including makespan, response time, energy consumption, execution cost, load balancing, resource utilization, and task completion rate [23,26]. Time-aware, deadline-constrained, and multi-objective formulations have improved the applicability of fog scheduling in latency-sensitive environments [23,26]. However, many studies still provide limited details on continuous-to-discrete task-node encoding, normalization of heterogeneous QoS metrics, weighted objective aggregation, infeasible assignment repair, and penalty-based handling of resource and deadline violations, which may reduce reproducibility and weaken fair comparison.

### 2.2. Heuristic, Metaheuristic, and Learning-Based Scheduling Methods

To solve the NP-hard IoT–Fog scheduling problem, heuristic, metaheuristic, and learning-based methods have been extensively investigated [26,27]. Heuristic methods usually offer low computational cost but may be trapped in locally optimal scheduling decisions. Population-based metaheuristics improve global search by maintaining multiple candidate solutions and have been applied to balance latency, energy, cost, and resource utilization [23,27]. More recently, reinforcement learning, deep learning, neuro-fuzzy learning, and adaptive scheduling frameworks have been introduced to enhance decision-making in dynamic fog environments [27,28]. Despite these advances, metaheuristic schedulers remain sensitive to parameter settings and may suffer from premature convergence, while learning-based methods often require careful reward design, sufficient training data, and additional computational overhead.

### 2.3. Hybrid Metaheuristics and Search Experience Reuse

Hybrid metaheuristics have become a common direction for improving IoT–Fog scheduling performance by combining complementary mechanisms such as global exploration, local exploitation, elite learning, chaotic initialization, Lévy-flight perturbation, adaptive parameter control, and pheromone-inspired selection [27,29]. These designs can enhance search capability, but many of them remain operator-centric, where performance improvement mainly depends on adding or modifying search operators. In such frameworks, high-quality solutions generated during evolution are often used only implicitly through the current best individual or elite replacement, rather than being explicitly extracted, archived, and reused as structured guidance sources. Therefore, the systematic management of quality sources and their reuse as multi-source guidance remain insufficiently explored in hybrid scheduling optimization.

### 2.4. Summary and Research Positioning

Overall, existing IoT–Fog scheduling studies have improved QoS-aware task allocation through advanced modeling, metaheuristic optimization, and learning-based decision-making [22,23,27]. However, three limitations remain evident: the optimization formulation is not always sufficiently transparent, population-based search may still be unstable in complex discrete scheduling spaces, and hybrid metaheuristics often lack explicit mechanisms for preserving and reusing high-quality search experience. To address these issues, ASQS is positioned as a quality-source-driven scheduling optimizer that combines explicit scheduling formulation with anchor-based experience preservation and multi-anchor synchronized search, aiming to improve search stability, diversity maintenance, and reproducibility in resource-constrained IoT–Fog scheduling.

## 3. Component Roles and Design Motivation of ASQS

The proposed ASQS framework is not a direct combination of several standalone metaheuristic algorithms. Instead, it reorganizes selected search principles into a quality-source-driven multi-anchor search framework. Specifically, the hierarchical population organization is used to extract quality sources from different fitness layers, the FNO-based operator provides directional search guidance, the Lévy-flight mechanism enhances escape ability, and the ACO-inspired rule is redesigned as a probabilistic synchronization strategy among anchors. In addition, the cooperative search principle, inspired by cooperative metaheuristic algorithms [30], is incorporated to coordinate information exchange among quality-source anchors and candidate schedules, thereby supporting collaborative exploration and exploitation within the unified ASQS framework.

Table 1 summarizes the role of each component and clarifies whether it is newly proposed or adapted from existing techniques.

### 3.1. Motivation of Quality-Source-Driven Multi-Anchor Search

Inspired by the hierarchical population organization mechanism of Hybrid Rice Optimization (HRO) [31], ASQS ranks candidate schedules by fitness and organizes them into different quality layers. This layered organization enables ASQS to extract representative quality-source anchors from different fitness regions rather than relying only on top-ranked solutions. Table 2 summarizes the role of each quality layer in anchor construction, where high-quality, medium-quality, and low-quality layers respectively provide exploitation-oriented, stability-oriented, and diversity-oriented sources for the subsequent multi-anchor synchronized search.

### 3.2. Roles of Adapted Search Operators

The global search strategy of ASQS draws on the directional guidance concept of the Farthest Better and Nearest Worse Optimizer (FNO), which relies on two contrastive references: the farthest better solution and the nearest worse solution [32]. In the global search stage, the farthest better reference encourages candidate schedules to move toward remote higher-quality regions, while the nearest worse reference helps reduce movements toward nearby inferior regions. This fitness-contrast-based guidance enables ASQS to extend the search scope in a more directed manner and is coordinated with the quality-source anchor archive during candidate updates.

ASQS adopts a probability-guided multi-anchor synchronization strategy motivated by the selection and feedback principles of Ant Colony Optimization (ACO) [33]. In this strategy, quality-source anchors are selected according to probabilities determined by their fitness-based importance and layer information. As a result, high-quality anchors provide stronger guidance for candidate updates, while medium- and low-quality anchors are also involved to preserve search diversity. This probability-guided synchronization helps coordinate information sharing among multiple anchors within the ASQS search process.

ASQS employs a Lévy-flight-inspired escape perturbation motivated by the heavy-tailed search behavior of Lévy flight [34]. This behavior produces frequent short-range perturbations together with occasional long-range jumps, enabling candidate schedules to move out of restricted search regions when the search progress slows down. The perturbation is applied conditionally within the ASQS search process and works in coordination with the quality-source anchor archive to help maintain exploration diversity.

## 4. Proposed ASQS Algorithm

### 4.1. Overall Framework of ASQS

ASQS is organized as a quality-source-guided closed-loop search framework. At each iteration, FNO-based directional search and fitness-adaptive Lévy-flight escape first expand the current population and form an intermediate candidate pool. Quality-source anchors are then extracted from this pool across different fitness layers to construct the anchor archive. The archive provides multi-anchor guidance for the ACO-inspired synchronization module, enabling candidate updates to exploit multiple promising scheduling regions instead of a single best solution. The synchronized candidates are finally evaluated and filtered through greedy selection to form the next population. Thus, the main components of ASQS are integrated as cooperative functional operators within a unified iterative procedure, rather than as independent optimizers.

### 4.2. Solution Encoding and Fitness Evaluation

To apply ASQS to fog task scheduling, each continuous candidate generated by the search operators must be decoded into a discrete task-node assignment before evaluation. This subsection defines the solution representation, decoding rule, scheduling metrics, constraint handling strategy, and greedy selection criterion used in ASQS.

In ASQS, the *p*th candidate solution is represented by a continuous vector $X_{p}=\left[x_{p,1},x_{p,2},\ldots ,x_{p,n}\right]$, where *n* is the number of IoT tasks and each dimension corresponds to one task. Before evaluation, $X_{p}$ is decoded into a discrete assignment vector $A_{p}=\left[a_{p,1},a_{p,2},\ldots ,a_{p,n}\right]$, where $a_{p,i}\in \left\{1,2,\ldots ,m\right\}$ denotes the fog node selected for task $T_{i}$, and *m* is the number of fog nodes. Each dimension is first clipped into [0,1], and the discrete node index is obtained as

(1) $$x_{p,i}=\min\left(1,\max\left(0,x_{p,i}\right)\right),\;a_{p,i}=\min\left(m,\max\left(1,\left\lceil x_{p,i}\cdot m\right\rceil \right)\right).$$

This decoding rule ensures that each task is assigned to exactly one fog node.

For a decoded schedule $A_{p}$, if task $T_{i}$ is assigned to fog node $F_{a_{p,i}}$, its transmission time, execution time, and basic processing time are calculated as

(2) $$TT_{p,i}=\frac{D_{i}}{B_{a_{p,i}}},\;ET_{p,i}=\frac{L_{i}}{C_{a_{p,i}}},\;PT_{p,i}=TT_{p,i}+ET_{p,i},$$

where $D_{i}$ and $L_{i}$ denote the input data size and computational workload of task $T_{i}$, respectively, and $B_{a_{p,i}}$ and $C_{a_{p,i}}$ denote the bandwidth and computing capacity of the assigned fog node. Tasks assigned to the same fog node are executed sequentially according to the task index order. Let $Q_{j}$ denote the accumulated finish time of fog node $F_{j}$, with $Q_{j}=0$ initially. The final completion time of task $T_{i}$ and the corresponding node update are defined as

(3) $$FT_{p,i}=Q_{a_{p,i}}+PT_{p,i},\;Q_{a_{p,i}}\leftarrow FT_{p,i}.$$

Based on the finish-time evaluation, the makespan, average service latency, and energy consumption of schedule $A_{p}$ are calculated as

(4) $$MS_{p}=\max_{1\leq j\leq m}Q_{j},\;LAT_{p}=\frac{1}{n}\sum_{i=1}^{n}FT_{p,i},\;E_{p}=\sum_{i=1}^{n}P_{a_{p,i}}\cdot ET_{p,i},$$

where $P_{a_{p,i}}$ is the power consumption rate of the assigned fog node. The latency is computed using $FT_{p,i}$ so that both processing time and waiting time are considered.

In addition to the above objective-related metrics, the deadline satisfaction rate and load-balance index are reported to evaluate schedule feasibility and workload distribution. The deadline satisfaction rate is defined as

(5) $$DSR_{p}=\frac{1}{n}\sum_{i=1}^{n}I\left(FT_{p,i}\leq DL_{i}\right),$$

where I(·) is an indicator function that returns 1 if the condition is satisfied and 0 otherwise. A larger $DSR_{p}$ indicates that a higher proportion of tasks are completed before their deadlines.

The load-balance index is calculated using the standard deviation of the final accumulated finish times of fog nodes:

(6) $$LB_{p}=\sqrt{\frac{1}{m-1}\sum_{j=1}^{m}{\left(Q_{j}-\bar{Q}\right)}^{2}},\;\bar{Q}=\frac{1}{m}\sum_{j=1}^{m}Q_{j},$$

where $Q_{j}$ denotes the final accumulated finish time of fog node $F_{j}$. A smaller $LB_{p}$ indicates a more balanced workload distribution among fog nodes.

Since these metrics have different scales, they are normalized before aggregation:

(7) $$MS_{p,norm}=\frac{MS_{p}}{MS_{ref}},\;E_{p,norm}=\frac{E_{p}}{E_{ref}},\;LAT_{p,norm}=\frac{LAT_{p}}{LAT_{ref}}.$$

In the simulation, $MS_{ref}=2\cdot \max\left(DL_{i}\right)$, $E_{ref}=\left(\sum_{j=1}^{m}P_{j}\right)\cdot \max\left(DL_{i}\right)$, and $LAT_{ref}=\max\left(DL_{i}\right)$. These reference values are fixed within each scheduling scenario and are used only for scale normalization. The normalized objective is defined as

(8) $$F_{obj}\left(A_{p}\right)=w_{1}MS_{p,norm}+w_{2}E_{p,norm}+w_{3}LAT_{p,norm},$$

where $w_{1}+w_{2}+w_{3}=1$ and $w_{1},w_{2},w_{3}\geq 0$.

The resource capacity violation and deadline violation are defined as

(9) $$V_{cap}\left(A_{p}\right)=\sum_{j=1}^{m}\max\left(0,\sum_{i:a_{p,i}=j}r_{i}-R_{j}\right),$$

and

(10) $$V_{ddl}\left(A_{p}\right)=\sum_{i=1}^{n}\max\left(0,FT_{p,i}-DL_{i}\right),$$

where $r_{i}$ is the resource demand of task $T_{i}$, $R_{j}$ is the resource capacity of fog node $F_{j}$, and $DL_{i}$ is the deadline of task $T_{i}$.

For reporting purposes, the overall constraint violation is calculated as

(11) $$V_{total}\left(A_{p}\right)=V_{cap}\left(A_{p}\right)+V_{ddl}\left(A_{p}\right).$$

The final fitness value is then given by

(12) $$Fitness\left(A_{p}\right)=F_{obj}\left(A_{p}\right)+\lambda_{1}V_{cap}\left(A_{p}\right)+\lambda_{2}V_{ddl}\left(A_{p}\right),$$

where $\lambda_{1}$ and $\lambda_{2}$ are penalty coefficients. A smaller fitness value indicates a better scheduling solution.

To further improve feasibility, a repair operation is applied when capacity violations occur. For an overloaded fog node, the task with the largest resource demand is migrated to a node with the smallest current resource load that can satisfy the capacity constraint. If no feasible node exists, the task is moved to the node with the smallest current resource load to reduce the overload degree. If the repair process cannot fully eliminate the violation within the maximum number of attempts, the remaining violation is still penalized in the fitness function.

After a new candidate is generated by an ASQS update operator, it is decoded, repaired if necessary, and evaluated using the above fitness function. The greedy selection criterion is defined as

(13) $$X_{p}\leftarrow \left\{\begin{array}{cc} X_{p}^{new}, & Fitness\left(A_{p}^{new}\right)<Fitness\left(A_{p}^{old}\right),\\ X_{p}^{old}, & \mathrm{otherwise}. \end{array}\right.$$

where $A_{p}^{new}$ and $A_{p}^{old}$ are the decoded assignment vectors corresponding to $X_{p}^{new}$ and $X_{p}^{old}$, respectively.

### 4.3. FNO-Based Search Operator

The FNO-based operator is used in ASQS to provide directional search guidance for candidate schedules [32]. For each candidate $X_{p}$, the farthest better solution $X_{p}^{FB}$ and the nearest worse solution $X_{p}^{NW}$ are identified according to fitness and distance. Specifically, $X_{p}^{FB}$ denotes the better solution with the largest distance from $X_{p}$, while $X_{p}^{NW}$ denotes the worse solution with the smallest distance from $X_{p}$.

In ASQS, the FNO-based directional update is implemented in a velocity-assisted form:

(14) $$V_{p}^{t+1}=wV_{p}^{t}+c_{1}r_{1}⊙\left(X_{p}^{FB}-X_{p}^{t}\right)+c_{2}r_{2}⊙\left(X_{p}^{t}-X_{p}^{NW}\right)+c_{3}r_{3}⊙\left(X_{\mathrm{best}}^{t}-X_{p}^{t}\right),$$

(15) $$X_{p}^{t+1}=X_{p}^{t}+V_{p}^{t+1}.$$

where $V_{p}^{t}$ is the velocity vector, $X_{\mathrm{best}}^{t}$ is the current global-best solution, $r_{1}$, $r_{2}$, and $r_{3}$ are random vectors sampled from [0,1], and ⊙ denotes element-wise multiplication. The inertia weight is set to w=0.9−0.5×ratio, where ratio=t/T, and the acceleration coefficients are set to $c_{1}=c_{2}=c_{3}=1.5$. The initial velocity is $V_{0}=0$.

The term associated with $X_{p}^{FB}$ guides the candidate toward promising distant regions, while the term associated with $X_{p}^{NW}$ helps avoid inferior neighboring regions. The global-best term further provides convergence guidance. In ASQS, this operator is not used as an independent optimizer but serves as a directional update component coordinated by the quality-source anchor archive.

### 4.4. Lévy-Flight Escape Mechanism

To enhance the ability of ASQS to escape from local optima, a Lévy-flight escape mechanism is incorporated after the FNO-based directional search. For each candidate, the escape operation is adaptively determined by an escape energy that combines its relative fitness gap with the remaining iteration ratio:

(16) $$E_{p}^{t}=\frac{\left|Fitness\left(A_{\mathrm{best}}^{t}\right)-Fitness\left(A_{p}^{t}\right)\right|}{\left|Fitness(A_{\mathrm{best}}^{t})\right|+\epsilon }\left(1-\frac{t}{T}\right),$$

where $Fitness(A_{p}^{t})$ is the fitness value of the decoded schedule corresponding to $X_{p}^{t}$, $Fitness(A_{\mathrm{best}}^{t})$ is the current best fitness value, *T* is the maximum number of iterations, and ϵ is a small positive constant used to avoid division by zero.

When $E_{p}^{t}\geq 1$, the *p*th candidate solution is perturbed by Lévy flight:

(17) $$X_{p}^{escape}=X_{p}^{t}+\lambda \cdot \mathrm{Levy}\left(\beta \right)⊙\left(X_{p}^{t}-X_{\mathrm{best}}^{t}\right),$$

where λ=0.01 is the perturbation scale, Levy(β) denotes a Lévy random step with the distribution parameter β=1.5, ⊙ represents element-wise multiplication, and $X_{\mathrm{best}}^{t}$ is the current best candidate solution. The threshold is fixed to 1 so that Lévy perturbation is mainly applied to candidates with a sufficiently large relative fitness gap, especially in the early search stage. After the escape operation, the generated candidate is clipped into the continuous search range, decoded into a discrete task-node assignment, repaired if necessary, and evaluated using the fitness function.

### 4.5. Quality-Source Extraction and Anchor Archive Construction

After the three-layer FNO-based search and Lévy-flight perturbation, the updated population contains valuable search information distributed across different quality layers. Relying only on the global-best solution may cause excessive concentration around a single region and increase the risk of premature convergence. Therefore, ASQS extracts representative elite solutions from multiple layers and organizes them into a quality-source anchor archive. These anchors act as reusable guidance centers for the subsequent ACO-inspired multi-anchor synchronized search.

Let $P_{t}^{\left(l\right)}$ denote the *l*th quality layer of the population at iteration *t*, where l=1,2,3 correspond to the high-quality, medium-quality, and low-quality layers, respectively. After the search and perturbation stages, a subset of representative solutions is selected from each layer according to fitness ranking. The anchor subset extracted from layer *l* is defined as

(18) $$A_{t}^{\left(l\right)}=\mathrm{TopK}\left(P_{t}^{\left(l\right)},K_{l}\right),\;l=1,2,3,$$

where $K_{l}$ is the number of anchors selected from layer *l*, and TopK(·) returns the top-ranked candidates with smaller fitness values. Compared with using only the best individual, this layer-wise extraction strategy preserves multiple promising directions and allows ASQS to reuse high-quality information discovered by different parts of the population.

The global anchor archive is constructed by merging the anchor subsets from all quality layers:

(19) $$A_{t}=A_{t}^{\left(1\right)}\cup A_{t}^{\left(2\right)}\cup A_{t}^{\left(3\right)}.$$

Here, anchors from the high-quality layer mainly provide exploitation-oriented guidance, anchors from the medium-quality layer support a balanced search transition, and anchors from the low-quality layer preserve alternative directions for diversity. Thus, the archive represents a multi-source guidance structure rather than a single elite solution.

To determine the contribution of different anchors in the subsequent synchronized search, each anchor is assigned a probability according to both its fitness ranking and its layer source. Let $M_{t}=\left|A_{t}\right|$ be the number of anchors in the archive and rank(k) be the fitness rank of the *k*th anchor, where rank(k)=1 indicates the best anchor. The ranking weight is defined as

(20) $$w_{r}\left(k\right)=\frac{1}{rank(k)}.$$

In addition, the layer-dependent weight is defined as

(21) $$w_{l}\left(k\right)=\left\{\begin{array}{cc} \omega_{1}, & A_{k}\in A_{t}^{\left(1\right)},\\ \omega_{2}, & A_{k}\in A_{t}^{\left(2\right)},\\ \omega_{3}, & A_{k}\in A_{t}^{\left(3\right)}, \end{array}\right.$$

where $\omega_{1}$, $\omega_{2}$, and $\omega_{3}$ control the relative influence of anchors from different layers. In this study, $\omega_{1}=1.0$, $\omega_{2}=0.7$, and $\omega_{3}=0.4$. In general, high-quality anchors are assigned larger weights to enhance exploitation, while medium- and low-layer anchors retain nonzero probabilities to maintain diversity.

The final selection probability of the *k*th anchor is calculated as

(22) $$\pi_{k}=\frac{w_{r}\left(k\right)w_{l}\left(k\right)}{\sum_{j=1}^{M_{t}}w_{r}\left(j\right)w_{l}\left(j\right)}.$$

This probability design enables ASQS to prefer high-quality anchors while still allowing other representative anchors to participate in the search. The archive is updated at each iteration after the layered search and perturbation stages so that the synchronized search can be guided by the latest quality sources discovered by the population.

### 4.6. Anchor-Seeding and ACO-Inspired Multi-Anchor Synchronized Search

ASQS introduces an ACO-inspired multi-anchor synchronization strategy to share useful scheduling patterns among candidate solutions. The positive-feedback and probabilistic selection principles of ACO are adapted to anchor-level guidance rather than edge-level path construction. In classical ACO, paths with stronger pheromone information are more likely to be selected and further reinforced. In ASQS, this idea is transferred to the quality-source anchor archive: anchors with better fitness ranking and stronger layer importance are assigned higher selection probabilities and are therefore more likely to serve as guidance sources for subsequent candidate generation.

Anchor seeding provides the mechanism by which the information stored in the archive is injected back into the population. Instead of forcing all individuals to follow the current global-best solution, each individual probabilistically selects one anchor from the archive constructed in Section 4.5. The selected anchor is used as a seed center for generating a synchronized candidate. Therefore, anchor seeding acts as the interface between quality-source extraction and ACO-inspired synchronization: the archive determines which scheduling patterns are reliable, the ACO-inspired probability model determines how likely each anchor is reused, and the synchronization operator determines how the selected anchor is perturbed and propagated to the population.

Suppose that the *k*th anchor $A_{t,k}$ is selected at iteration *t*, and its quality-layer index is *l*. A synchronized candidate for the *p*th solution is generated around the selected anchor as

(23) $$X_{p}^{sync}=A_{t,k}+s_{\tau }\left(l\right)\times \tau_{t}\times \mathrm{randn}\left(1,dim\right)+s_{a}\left(l\right)\times drift_{p}.$$

where $X_{p}^{sync}$ is the synchronized candidate, $A_{t,k}$ is the selected anchor seed, $s_{\tau }\left(l\right)$ controls the layer-dependent local sampling strength, randn(1,dim) is a Gaussian random vector, $s_{a}\left(l\right)$ controls the global-best drift strength, and $drift_{p}$ denotes the global guidance direction for the *p*th solution.

The first term in Equation (23) provides the anchor seed, which preserves the scheduling pattern extracted from a quality-source solution. The second term performs local stochastic sampling around the selected anchor. Its strength is adjusted by the layer-dependent factor $s_{\tau }\left(l\right)$ so that anchors from higher-quality layers can be exploited with a smaller search radius, while anchors from lower-quality layers can maintain a larger exploratory range. The third term introduces a weak drift toward the current global-best region, which provides directional guidance without making the search completely dependent on a single best solution.

The sampling radius is adaptively reduced as

(24) $$\tau_{t}=0.1\times searchRange\times \left(1-ratio\right),\;ratio=\frac{t}{T},\;searchRange=\left(UB-LB\right)$$

where UB and LB are the upper and lower bounds of the continuous search space, and *T* is the maximum number of iterations. A larger $\tau_{t}$ in the early stage allows broader exploration around multiple anchors, whereas a smaller $\tau_{t}$ in the later stage promotes refined exploitation near promising anchor regions.

After generation, $X_{p}^{sync}$ is clipped, decoded, repaired if necessary, and evaluated using the fitness function. The synchronized candidate is accepted only if it improves the current solution. Through this anchor-seeding-based synchronization strategy, ASQS reuses high-quality scheduling patterns through ACO-inspired probabilistic selection, while still preserving diversity by allowing different individuals to select different anchors. As a result, the population can exploit multiple promising scheduling regions simultaneously, improving information sharing and reducing dependence on a single global-best solution.

### 4.7. Overall Procedure of ASQS

This subsection integrates the components introduced in Section 4.1, Section 4.2, Section 4.3, Section 4.4, Section 4.5 and Section 4.6 into a unified execution procedure. Given the task set, fog-node set, search-space bounds, objective weights, constraint parameters, and algorithmic control parameters, ASQS aims to obtain the best task-node schedule and its associated performance indicators.

**Figure 2:**
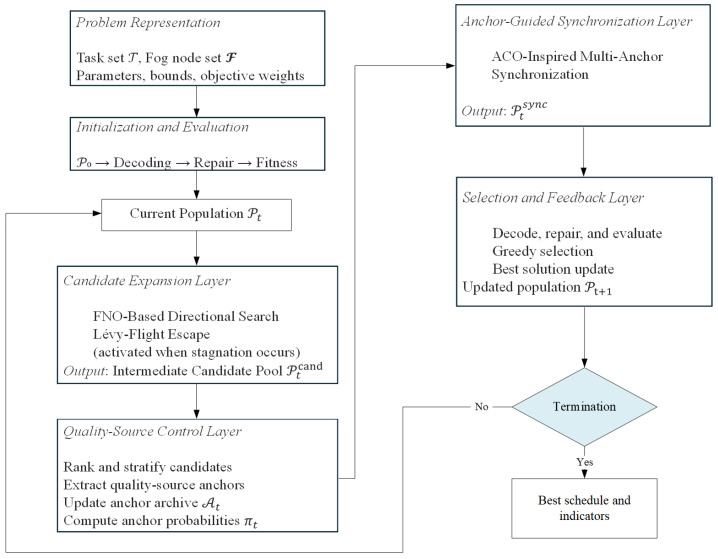
Quality-source-guided closed-loop architecture of ASQS.

As summarized in Algorithm 1 and Figure 2, ASQS first initializes a population of continuous candidate solutions and maps them into task-node assignments through decoding, feasibility repair, and fitness evaluation. During each iteration, the population is ranked and stratified according to fitness quality, from which quality-source anchors are extracted to update the anchor archive. Guided by the current population state and the anchor archive, ASQS generates candidate schedules through FNO-based directional search, fitness-adaptive Lévy-flight escape, and ACO-inspired multi-anchor synchronization. The generated candidates are subsequently decoded, repaired, evaluated, and compared with the current solutions through greedy selection. The best solution is updated iteratively until the maximum number of iterations is reached.

The computational cost of ASQS is mainly dominated by candidate decoding, feasibility repair, and fitness evaluation. With population size *N* and maximum iteration number *T*, the overall complexity can be approximately expressed as $O(T\cdot N\cdot C_{eval})$, where $C_{eval}$ denotes the cost of evaluating one candidate schedule and is primarily determined by the task and fog-node scales.

**Algorithm 1** Overall procedure of ASQS
  1:**Input:** Task set T, fog node set F, population size *N*, maximum iterations *T*, bounds LB and UB, objective weights, constraint parameters, and ASQS control parameters  2:**Output:** Best schedule $A_{\mathrm{best}}$, best fitness value $fit_{\mathrm{best}}$, and scheduling indicators  3:Initialize population $P_{0}={\left\{X_{p}^{0}\right\}}_{p=1}^{N}$ within [LB,UB]  4:Evaluate $P_{0}$ by decoding, repairing, and fitness calculation  5:Initialize $A_{\mathrm{best}}$, $X_{\mathrm{best}}$, and $fit_{\mathrm{best}}$  6:**for** t=0 to T−1 **do**  7:    Generate FNO-based candidates: $P_{t}^{FNO}\leftarrow FNOSearch\left(P_{t}\right)$  8:    Evaluate $P_{t}^{FNO}$  9:    Generate Lévy-escape candidates according to $E_{p}^{t}$: $P_{t}^{Levy}\leftarrow LevyEscape\left(P_{t}^{FNO}\right)$10:    Evaluate $P_{t}^{Levy}$11:    Form intermediate candidate pool $P_{t}^{cand}\leftarrow P_{t}\cup P_{t}^{Levy}$12:    Construct anchor archive and probabilities: $\left(A_{t},\pi_{t}\right)\leftarrow BuildAnchorArchive\left(P_{t}^{cand}\right)$13:    Generate synchronized candidates: $P_{t}^{sync}\leftarrow AnchorSync\left(P_{t},A_{t},\pi_{t}\right)$14:    Evaluate $P_{t}^{sync}$15:    Update population: $P_{t+1}\leftarrow GreedySelect\left(P_{t},P_{t}^{sync}\right)$16:    Update $A_{\mathrm{best}}$, $X_{\mathrm{best}}$, and $fit_{\mathrm{best}}$17:
**end for**
18:Calculate scheduling indicators of $A_{\mathrm{best}}$19:**return** $A_{\mathrm{best}}$, $fit_{\mathrm{best}}$, and scheduling indicators


## 5. Experiments and Discussion

To comprehensively evaluate the proposed algorithm, a series of experiments are conducted, including benchmark-function optimization, sensitivity analysis, ablation studies, IoT–Fog task scheduling experiments, statistical significance tests, and large-scale task-intensive scalability analysis. Specifically, 33 benchmark functions are used to test the general optimization ability and convergence stability of ASQS. Sensitivity analysis and ablation studies are conducted to examine parameter robustness and component effectiveness. IoT–Fog scheduling experiments are further designed under node-scaling, task-scaling, and large-scale task-intensive scenarios, including 100 fog nodes and up to 8000 IoT tasks. The algorithms are compared using mean fitness, best fitness, standard deviation, makespan, energy consumption, average latency, deadline satisfaction rate, load balance, runtime, and constraint violation, thereby evaluating scheduling efficiency, QoS satisfaction, workload balance, constraint feasibility, and scalability.

### 5.1. Experiment I: Global Optimization with a Set of 33 Benchmark Mathematical Functions

#### 5.1.1. Benchmark Functions

The 33 benchmark functions are grouped into four categories with increasing landscape complexity. F01–F08 are unimodal functions for evaluating exploitation and convergence accuracy; F09–F20 are multimodal functions for testing exploration and local-optimum avoidance; F21–F26 are hybrid functions for assessing the balance between exploration and exploitation in coupled landscapes; and F27–F33 are composition functions for examining robustness on highly irregular and deceptive search spaces. Their detailed definitions are provided in Table 3, Table 4, Table 5 and Table 6.

#### 5.1.2. Performance Evaluation Metrics

To evaluate the optimization accuracy, stability, and statistical significance of the compared algorithms, the experimental results are analyzed using the mean value (Avg), standard deviation (Std), ranking, Wilcoxon signed-rank test, and effect size. For *M* independent runs, Avg and Std are calculated as follows:

(25) $$\mathrm{Avg}=\frac{1}{M}\sum_{i=1}^{M}f_{i},$$

(26) $$\mathrm{Std}=\sqrt{\frac{1}{M-1}\sum_{i=1}^{M}{\left(f_{i}-\mathrm{Avg}\right)}^{2}},$$

where $f_{i}$ is the fitness value obtained in the *i*th run. Algorithms are ranked according to their average fitness values, and lower values indicate better performance for minimization problems. The Wilcoxon signed-rank test is conducted at a significance level of α=0.05 to assess whether the performance differences are statistically significant. In addition, Better/Equal/Worse counts and effect sizes are reported to summarize pairwise comparison results and quantify the magnitude of performance differences.

#### 5.1.3. Comparison with Other Algorithms and Parameter Setting

Table 7 summarizes the parameter settings used in the benchmark-function experiments, and Table 8 presents the comparative results. The selected baselines include classical metaheuristics, such as FNO [32], ACO [33], HSA [34], DE [35], GWO [36], and WOA [37]; advanced evolutionary and hybrid optimizers, including MetaDE [38], HPSOBOA [39], PS-BES [40], and PSOBOA [41]; and the composite metaheuristic algorithm CMA. These competitors represent diverse optimization mechanisms rather than a narrow set of similar algorithms, making the comparison more stringent.

To avoid biased conclusions caused by unequal configurations, all algorithms were tested under the same dimension, population size, number of runs, and computational budget. Baseline parameters follow the original literature or standard settings, while ASQS uses one fixed configuration throughout the benchmark experiments. No algorithm was given additional iterations or a larger population. Thus, the reported differences mainly reflect the effectiveness of the search mechanisms rather than differences in experimental resources.

#### 5.1.4. Data Analysis

Table 8 compares ASQS with 12 competing algorithms on 33 benchmark functions. ASQS obtains the best Avg_Rank of 3.4 and ranks first overall, demonstrating the strongest aggregate performance among all methods. It achieves the best results on 10 functions, namely F3, F9, F11, F12, F17, F20, F21, F30, F31, and F32, and remains competitive on most of the remaining functions. This confirms that the advantage of ASQS is not restricted to a small subset of test cases.

The superiority of ASQS is especially evident on hybrid and composition functions, such as F21, F30, F31, and F32, where the search landscapes are more complex and deceptive. The result on F32 is particularly representative: ASQS obtains the best average value with zero standard deviation, indicating both high accuracy and strong stability. These results support the effectiveness of the quality-source archive and multi-anchor synchronized search in maintaining reliable guidance under complex optimization conditions.

ASQS does not dominate every function. ACO performs better on F14, F15, and F28; DE shows stronger results on F18, F19, F22, F23, F26, and F27; and PS-BES achieves the best ranks on F7, F8, F24, F25, F29, and F33. This indicates that some baseline algorithms remain advantageous on specific landscape structures. However, ASQS delivers the best overall ranking, showing a more balanced and robust performance across the entire benchmark suite.

#### 5.1.5. Significance Analysis

According to the Wilcoxon signed-rank test results presented in Table 9, the performance differences between ASQS and each competing algorithm were evaluated on 33 benchmark functions at a significance level of 0.05. The terms Better, Equal, and Worse denote the numbers of functions on which ASQS performs better than, equal to, or worse than the compared algorithm, respectively. The *p*-value is used to measure statistical significance, while the Symbol column indicates the final comparison result.

As shown in Table 9, ASQS obtains more Better results than most competing algorithms. Compared with HSA, FNO, PSO, GWO, and MetaDE, the Better/Equal/Worse results are 32/0/1, 30/1/2, 29/0/4, 28/0/5, and 28/2/3, respectively. ASQS also performs competitively against WOA, HPSOBOA, and PE-BES. Compared with CMA and DE, ASQS still shows advantages, although the margins are relatively smaller. For ACO and PSOBOA, the results are comparatively close, indicating comparable performance between these algorithms and ASQS on some benchmark functions.

All *p*-values are below 0.05, indicating that the differences between ASQS and all competing algorithms are statistically significant. The effect sizes are high when ASQS is compared with PSO and HSA, and are also notable against GWO, WOA, CMA, ACO, FNO, DE, and PE-BES. For MetaDE, HPSOBOA, and PSOBOA, the effect sizes are moderate, indicating that ASQS still retains an advantage, although the improvement is relatively limited.

The results in Table 9 confirm that ASQS is statistically superior to most competing algorithms and demonstrates strong practical performance across the 33 benchmark functions.

#### 5.1.6. Characteristic Analysis

**Figure 3:**
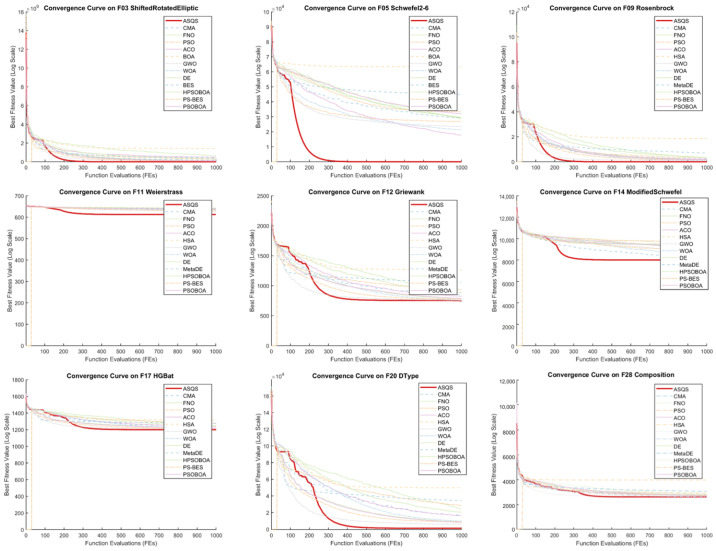
Representative convergence curves of ASQS and competing algorithms on selected benchmark functions.

Figure 3 presents the convergence curves of ASQS and the competing algorithms on nine representative benchmark functions. Overall, ASQS shows competitive convergence behavior and reaches lower final fitness values on most displayed cases, which is consistent with the ranking results in Table 8. In particular, ASQS exhibits rapid descent on F05, F09, F12, F14, and F20, while several baseline algorithms converge more slowly or stagnate at higher fitness values. This indicates that ASQS can maintain effective search progress across different benchmark landscapes.

The observed convergence pattern is mainly attributed to the coordinated search structure of ASQS. The quality-layer organization maintains candidate solutions from different fitness levels, the FNO-based update provides directional search guidance, and the Lévy-flight perturbation helps escape stagnant regions. In addition, the quality-source archive and multi-anchor synchronized search allow the population to exploit multiple promising regions rather than relying on a single best solution. Consequently, ASQS achieves a stable balance between exploration and exploitation, leading to competitive convergence performance.

#### 5.1.7. Sensitivity Analysis

To justify the default parameter configuration of ASQS, we conducted a one-factor-at-a-time sensitivity analysis on four key parameters: the Lévy scale λ, the synchronization radius $\tau_{0}$, the layer scale $s_{\tau }$, and the attraction scale α. Three representative benchmark functions, F03, F12, and F30, were used to cover unimodal, multimodal, and composition landscapes.

**Figure 4:**
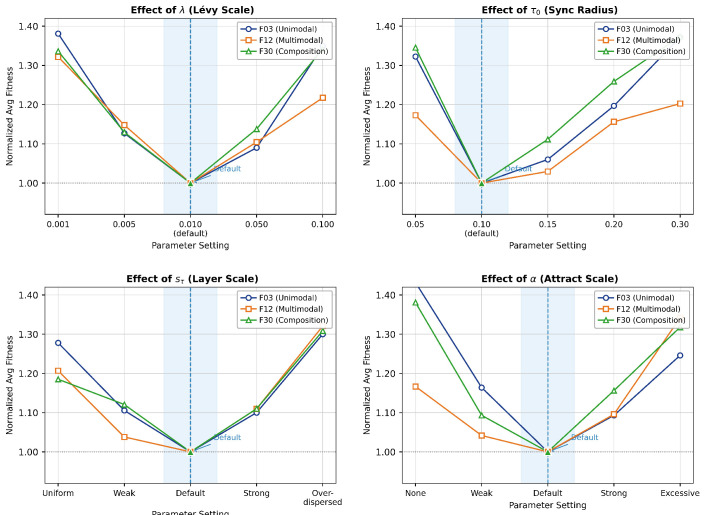
Sensitivity analysis of key ASQS parameters on representative benchmark functions. The normalized average fitness is reported for the Lévy perturbation scale λ, synchronization radius $\tau_{0}$, layer scale strategy $s_{\tau }$, and attraction scale α. The default setting of each parameter is normalized to 1.0.

Figure 4 and the rank results show a clear pattern: the default setting achieves the lowest normalized average fitness for every parameter and ranks first on all three benchmark functions. Specifically, λ=0.010, $\tau_{0}=0.10$, the default-layered $s_{\tau }$, and the default α all obtain an average rank of 1.00. This demonstrates that the adopted parameter configuration is the best-performing choice among the tested alternatives.

The performance degradation away from the default settings is also consistent. Too small λ weakens local-optimum escape, while too large λ causes unstable jumps. Too small $\tau_{0}$ limits anchor synchronization, whereas too large $\tau_{0}$ leads to over-synchronization. Uniform or over-dispersed $s_{\tau }$ settings damage the balance between exploration and exploitation. Removing or excessively increasing α weakens guidance or causes premature concentration.

### 5.2. Experiment II: Ablation Study of ASQS

**Figure 5:**
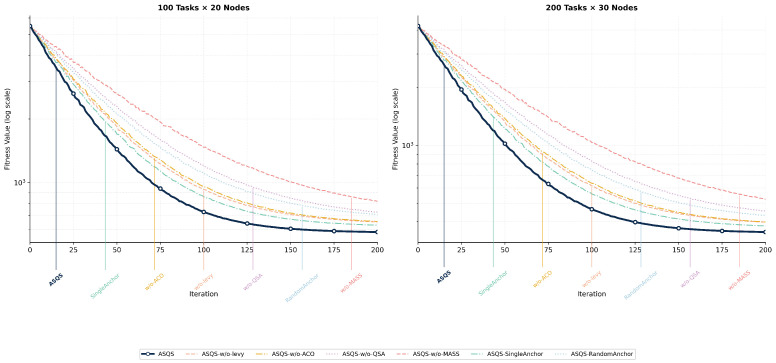
Convergence curves of ablation study.

**Figure 6:**
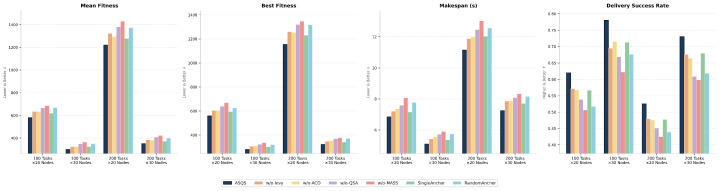
Ablation study—performance metrics comparison.

To further evaluate the contribution of each key component in the proposed ASQS, an extended ablation study was conducted. The complete ASQS was compared with six ablated variants, namely ASQS-w/o-Levy, ASQS-w/o-ACO, ASQS-w/o-QSA, ASQS-w/o-MASS, ASQS-SingleAnchor, and ASQS-RandomAnchor. Specifically, ASQS-w/o-Levy removes the Levy-based perturbation strategy, ASQS-w/o-ACO removes the ACO-based exploitation component, ASQS-w/o-QSA removes the quality-source anchor archive, ASQS-w/o-MASS removes the multi-anchor synchronized search mechanism, ASQS-SingleAnchor uses only a single best anchor for search guidance, and ASQS-RandomAnchor randomly selects anchors instead of using quality-guided anchor selection. The ablation experiments were performed under four IoT–Fog scheduling scenarios, including 100 tasks with 20 fog nodes, 100 tasks with 30 fog nodes, 200 tasks with 20 fog nodes, and 200 tasks with 30 fog nodes. To provide a comprehensive evaluation, both optimization-oriented metrics and practical scheduling metrics were considered, including mean fitness, best fitness, makespan, energy consumption, average latency, delivery success rate, load balance, runtime, and convergence behavior. The detailed numerical results are reported in Table 10 and Table 11, while the metric-level comparison and convergence behavior are illustrated in Figure 5 and Figure 6, respectively.

Figure 5 shows the convergence behavior of ASQS and its variants under two representative scenarios. ASQS consistently achieves the lowest fitness curve and the best final solution, indicating superior convergence efficiency and optimization capability. In contrast, all ablated variants converge to higher fitness values, confirming that removing any key component weakens the search performance. Notably, ASQS-w/o-MASS exhibits the poorest convergence, highlighting the critical role of multi-anchor synchronized search. The inferior results of ASQS-w/o-QSA and ASQS-RandomAnchor further verify the importance of quality-source anchor preservation and quality-guided anchor selection. These results demonstrate that the advantage of ASQS comes from the coordinated contribution of its core components.

Figure 6 further compares ASQS and its variants in terms of mean fitness, best fitness, makespan, and delivery success rate. ASQS achieves the lowest mean fitness, best fitness, and makespan across all scenarios, while obtaining the highest delivery success rate. This confirms that ASQS improves not only the objective value but also the practical scheduling quality. The consistent degradation of all ablated variants indicates that each removed component contributes to the overall performance. In particular, ASQS-w/o-MASS and ASQS-w/o-QSA show clear performance losses, demonstrating the necessity of multi-anchor synchronized search and quality-source anchor guidance.

Figure 6 and Table 10 provide consistent evidence for the overall effectiveness of ASQS. The complete ASQS achieves the lowest mean fitness, best fitness, makespan, and energy consumption across all four scenarios, while also obtaining the highest delivery success rate. This indicates that ASQS improves both the optimization objective and practical scheduling quality. All ablated variants show performance degradation, confirming that the superiority of ASQS comes from the cooperation of its key components rather than from a single operator. The marked decline of ASQS-w/o-MASS proves the critical role of multi-anchor synchronized search, while the weaker results of ASQS-w/o-QSA and ASQS-RandomAnchor verify the importance of quality-source anchor preservation and quality-guided anchor selection. Moreover, the gap between ASQS and ASQS-SingleAnchor demonstrates that multi-anchor cooperation provides more reliable guidance than single-anchor search.

Table 11 further evaluates ASQS from the perspective of practical scheduling metrics. ASQS achieves better average latency, delivery success rate, and load balance than its ablated variants, showing that its fitness improvement can be translated into more reliable and balanced IoT–Fog scheduling decisions. Although ASQS introduces a slightly higher runtime than some simplified variants, this overhead is reasonable because the complete method maintains the anchor archive, synchronizes multiple anchors, and performs enhanced search operations. Considering its consistent gains in solution quality, deadline satisfaction, and resource utilization, the additional computational cost is acceptable.

### 5.3. Experiment III: Fog Computing Task Scheduling Experiments

To evaluate the practical effectiveness of ASQS in fog computing task scheduling, two groups of experiments were designed under different task–node configurations. The detailed simulation settings are summarized in Table 12. In Group 1, the number of IoT tasks was fixed at 200, while the number of fog nodes increased from 5 to 25, generating five scenarios denoted as G1_N1 to G1_N5. In Group 2, the number of fog nodes was fixed at 10, while the number of IoT tasks increased from 50 to 500, generating five scenarios denoted as G2_T1 to G2_T5. These two groups were designed to examine the behavior of the scheduling algorithms under both node-scaling and task-scaling conditions.

In the simulation environment, task lengths, data sizes, deadlines, and resource demands were randomly generated within predefined ranges, while fog-node CPU capacities, bandwidths, resource capacities, and power values were also generated according to the settings in Table 12. To improve reproducibility and ensure a fair comparison, all algorithms were evaluated under the same generated task sets, fog-node configurations, and predefined random seeds. Each algorithm was independently executed 30 times, with a maximum of 200 iterations and a population size of 30.

ASQS was compared with nine representative scheduling algorithms, including classical heuristics, metaheuristic methods, and recent learning-based schedulers: GA-PSO [42,43], QHS [44], ACO-PSO [42,45], RWHS [44], SJF [46], HACO [45,47], MinMin [48], EcoTaskSched [2], and NF-MORL [13]. The performance of all algorithms was evaluated in terms of mean fitness, makespan, energy consumption, average latency, deadline satisfaction rate (DSR), load balance, runtime, and constraint violation.

#### 5.3.1. Evaluation Metrics

The evaluation metrics used in the experiments follow the definitions in Section 4.2. Specifically, makespan, average latency, energy consumption, deadline satisfaction rate, load balance, capacity violation, deadline violation, and final fitness are computed according to Equations (4)–(12). The reported constraint violation corresponds to the unweighted sum of capacity and deadline violations, as defined in Equation (11), whereas the fitness value includes the normalized objective and the weighted penalty terms.

For statistical comparison, Mean Fitness, Best Fitness, and Std Fitness are calculated over repeated independent runs. Lower Mean Fitness and Best Fitness indicate better scheduling quality, while lower Std Fitness reflects higher stability. Makespan, energy consumption, average latency, load balance, runtime, and constraint violation are minimized, whereas DSR is maximized. These metrics jointly evaluate solution quality, completion efficiency, energy cost, deadline feasibility, workload distribution, and computational overhead.

#### 5.3.2. Algorithm Parameter Settings

To ensure a fair and reproducible comparison, the parameter settings of the proposed ASQS and all comparative algorithms are summarized in Table 13. For all stochastic algorithms, the population size was set to nPop=30, and each algorithm was independently executed for numRuns=30 runs on each scheduling scenario. The problem dimension dim varies with the number of IoT tasks in each fog scheduling instance. Deterministic heuristics, including SJF and Min-Min, do not require population-based parameters and therefore use no additional stochastic configuration.

For ASQS, the number of populations is set to numPopulations=3, corresponding to the three quality-source anchor layers. The inertia weight follows w=0.9−0.5×ratio, where ratio denotes the normalized iteration progress. The attraction and repulsion coefficients are set to $c_{1}=c_{2}=c_{3}=1.5$, while the Lévy flight parameter is set to β=1.5. The perturbation coefficient is fixed as λ=0.01, and the pheromone diffusion radius is defined as τ=0.1×searchRange×(1−ratio). The multi-anchor diffusion scale factors and attraction drift factors are set to $s_{\tau }=\left[0.6,1.0,1.4\right]$ and $s_{a}=\left[0.2,0.3,0.4\right]$, respectively. The initial velocity is set to $V_{0}=0$. Unless otherwise specified, the symbols and parameter values used in the experiments follow Table 13.

#### 5.3.3. Convergence Analysis

**Figure 7:**
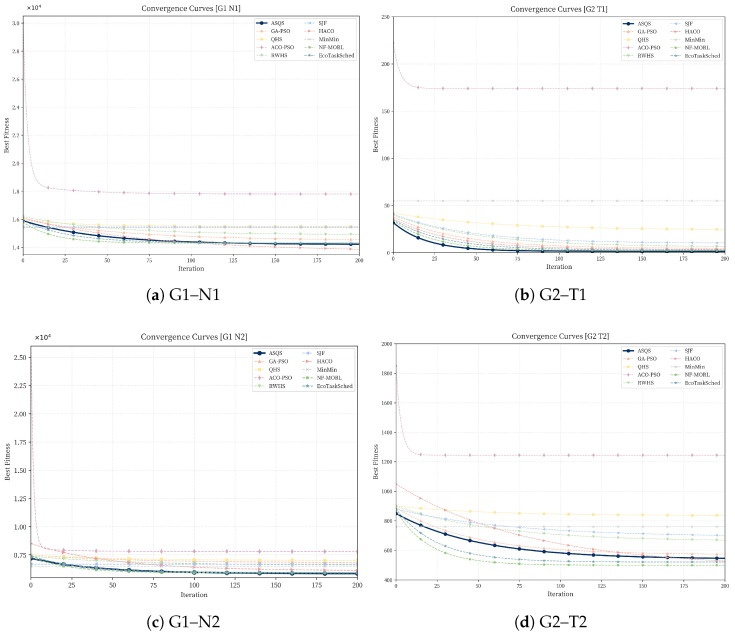
Convergence curves of ASQS and comparison algorithms under the first four test scenarios: (**a**) G1–N1, (**b**) G2–T1, (**c**) G1–N2 and (**d**) G2–T2. For visual clarity, ASQS is highlighted with a darker solid line, whereas the other algorithms are represented by lighter dashed lines.

**Figure 8:**
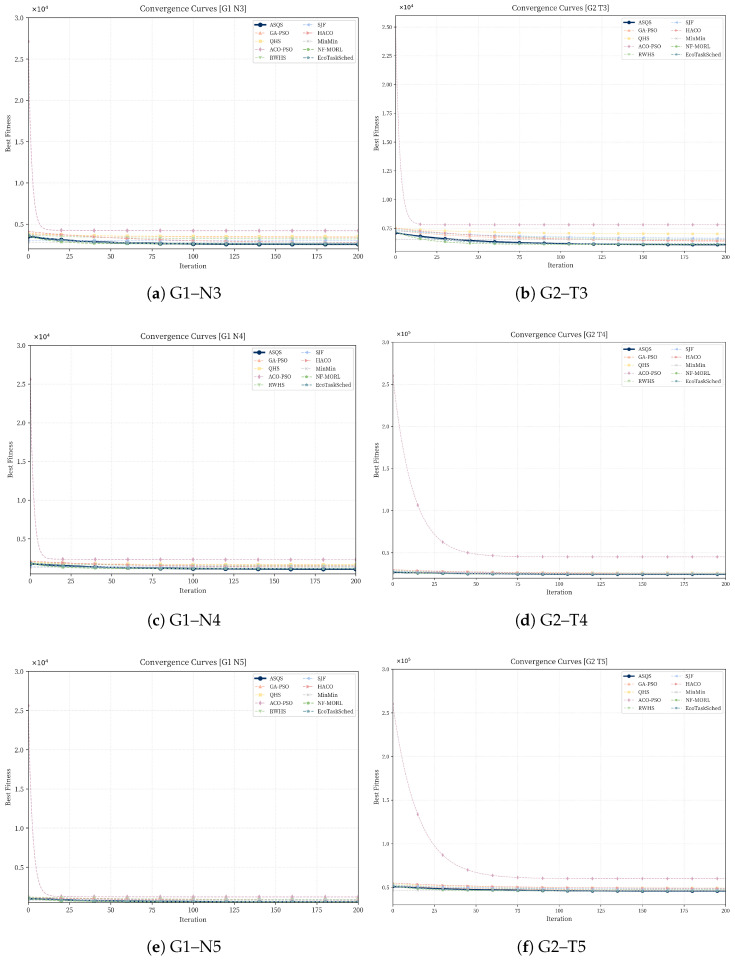
Convergence curves of ASQS and comparison algorithms under the remaining six test scenarios: (**a**) G1–N3, and (**b**) G2–T3, (**c**) G1–N4, (**d**) G2–T4, (**e**) G1–N5, and (**f**) G2–T5.

Figure 7 and Figure 8 present the best-so-far fitness trajectories of ASQS and the comparison algorithms. The curves are used to examine the search process, including early-stage descent, late-stage refinement, and premature stagnation. The observations in this subsection are therefore limited to convergence behavior, while the final scheduling quality is analyzed later using multiple performance indicators.

In the G1 scenarios, ASQS generally follows a low and smooth trajectory. In G1–N1, several competitive methods converge to close final fitness levels, and ASQS reaches the low-fitness region without noticeable oscillation. From G1–N2 to G1–N5, ASQS remains near the lower envelope of the convergence curves, particularly during the middle and late iterations. Compared with GA-PSO, QHS, SJF, and HACO, ASQS usually obtains a lower final fitness. However, NF-MORL, EcoTaskSched, RWHS, MinMin, and ASQS form a competitive group in several cases, indicating that ASQS demonstrates competitive rather than uniformly dominant convergence behavior.

ACO-PSO exhibits a different pattern. It decreases sharply at the beginning but then stagnates at a much higher fitness level in most scenarios. This suggests a clear tendency toward premature convergence. By contrast, ASQS shows a more sustained improvement process, which is consistent with the intended role of the quality-source archive and multi-anchor synchronization mechanism.

In the G2 scenarios, the relative behavior among the leading algorithms is more mixed. In G2–T1, ASQS has the steepest early decline and reaches the lowest final fitness. In G2–T2, NF-MORL obtains a lower final fitness than ASQS, although ASQS still outperforms most conventional baselines. In G2–T3 to G2–T5, ASQS stays within the leading convergence group together with NF-MORL, EcoTaskSched, RWHS, and MinMin. In G2–T4 and G2–T5, the large fitness values of ACO-PSO stretch the vertical axis, making the differences among the leading methods less visually distinguishable. Therefore, the exact ranking in these cases should be interpreted together with the numerical results.

From the convergence-curve perspective, ASQS avoids the high-level stagnation observed in ACO-PSO and maintains a stable best-so-far improvement trend in most scenarios. Nevertheless, these curves only describe the optimization trajectory and do not alone establish overall scheduling superiority. The subsequent statistical comparisons further evaluate the algorithms using fitness-related statistics and task-scheduling metrics.

#### 5.3.4. Multi-Metric Analysis Under Node-Scaling Scenarios

**Figure 9:**
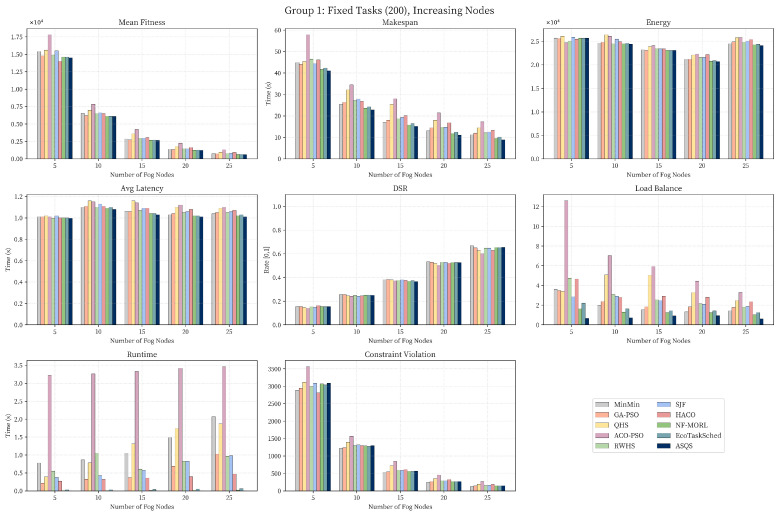
Multi-metric results under Group 1 node-scaling scenarios.

Figure 9 and Table 14 and Table 15 summarize the multi-metric evaluation results for the G1 scenarios. For readability, Table 14 reports the fitness-related statistics, makespan, and energy consumption, whereas Table 15 reports average latency, deadline satisfaction rate, load balance, runtime, and constraint violation. The analysis focuses on whether the algorithms can translate increased fog-resource availability into practical scheduling gains.

The node-scaling results reveal a clear resource-adaptive behavior of ASQS. As the number of fog nodes increases from 5 to 25, the mean fitness of ASQS decreases from $1.45\times 10^{4}$ to $6.09\times 10^{2}$, while the makespan is reduced from $4.10\times 10^{1}$ to 8.87. Meanwhile, its constraint violation decreases from $3.09\times 10^{3}$ to $1.47\times 10^{2}$, and its DSR increases from $1.55\times 10^{-1}$ to $6.55\times 10^{-1}$. These coordinated improvements indicate that ASQS can convert additional fog resources into lower scheduling cost, shorter completion time, and improved feasibility, rather than merely benefiting from the enlarged search space at the fitness level.

The advantage of ASQS is further reflected in its balanced performance profile. It obtains the best mean fitness and best fitness in four out of five node configurations, and achieves the lowest makespan, average latency, load-balance value, and runtime in all five configurations. This pattern suggests that ASQS improves scheduling efficiency without inducing severe load concentration or excessive computational overhead. Compared with NF-MORL and EcoTaskSched, ASQS also shows a more favorable trade-off between solution quality and decision-making cost, especially in larger-node cases.

It should be noted that comprehensive scheduling performance is not equivalent to single-metric dominance. ASQS does not always achieve the highest DSR or the lowest constraint violation, and ACO-PSO obtains the lowest energy consumption in the 5-node case. However, these metric-specific advantages are not consistently associated with better overall schedule quality, as they are often accompanied by longer makespan, poorer load balance, or higher runtime. Therefore, the G1 results indicate that ASQS is more advantageous in providing multi-dimensional scheduling robustness under node-scaling conditions.

Taken together, the node-scaling experiments suggest that ASQS can effectively adapt to variations in fog-resource availability. Its main advantage lies in transforming additional fog nodes into coordinated improvements in scheduling cost, completion efficiency, latency reduction, load distribution, and decision-making overhead. This performance profile makes ASQS suitable for fog computing scenarios where the scheduler must maintain efficient and stable task assignments under varying resource scales.

#### 5.3.5. Multi-Metric Analysis Under Task-Scaling Scenarios

**Figure 10:**
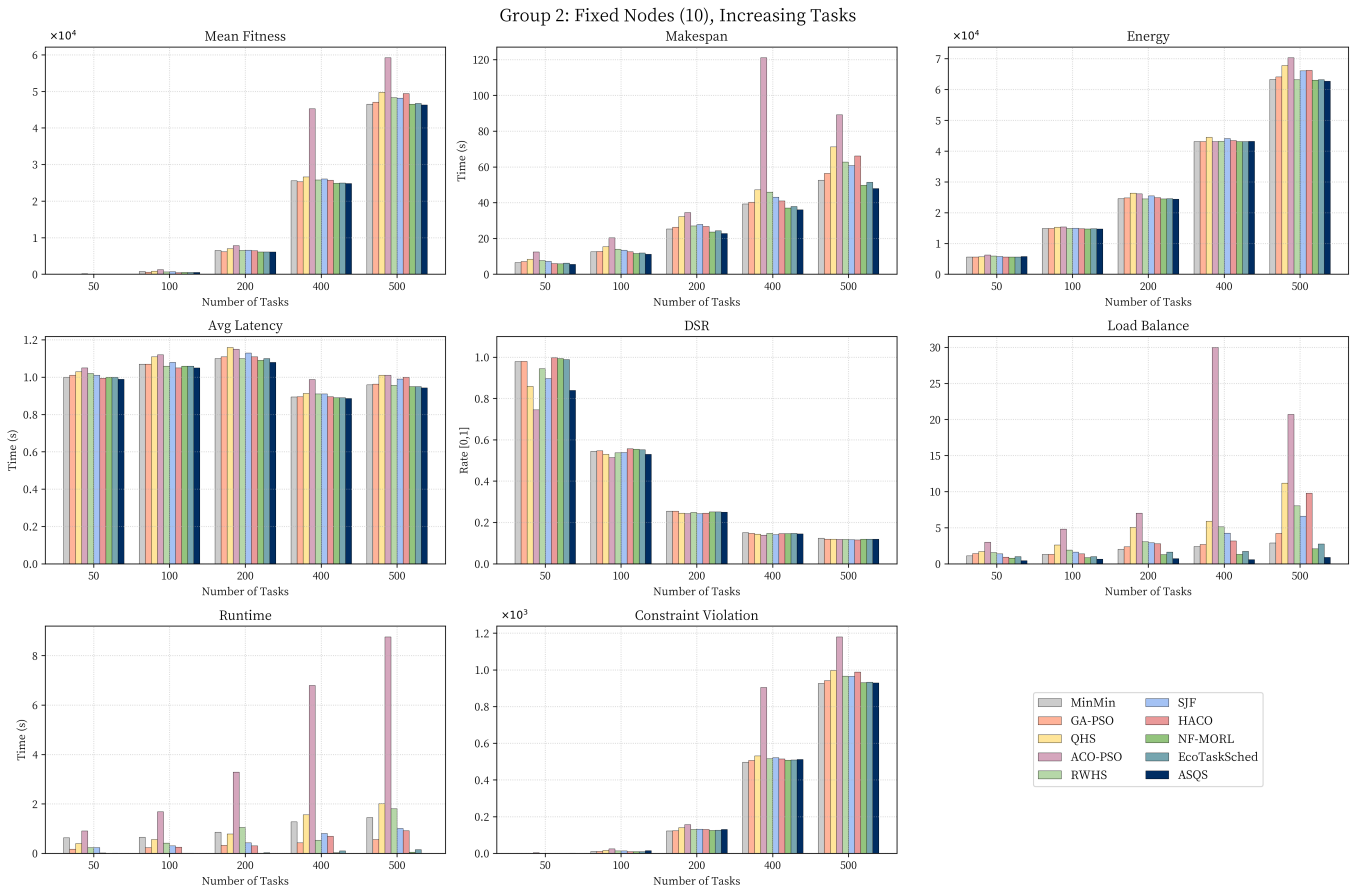
Multi-metric statistical results of ASQS and comparison algorithms under the Group 2 task-scaling scenarios with 10 fixed fog nodes and increasing IoT tasks.

The G2 scenarios examine the workload robustness of the algorithms when the fog-resource capacity is fixed and the number of IoT tasks increases. As shown in Figure 10 and Table 16 and Table 17, the increasing task load generally leads to higher fitness, makespan, energy consumption, and constraint violation, indicating a progressively more congested scheduling space.

Under this workload pressure, ASQS shows a stronger capability to restrain performance degradation. In the medium- and high-load cases with 200, 400, and 500 tasks, ASQS achieves the lowest mean fitness values of $6.08\times 10^{3}$, $2.48\times 10^{4}$, and $4.63\times 10^{4}$, respectively. More importantly, it consistently obtains the lowest makespan and average latency across all task scales, suggesting that ASQS better preserves completion efficiency as task demand increases.

The load-balance and runtime results further support this conclusion. ASQS achieves the lowest load-balance value in all G2 scenarios, indicating that its lower completion time is not obtained through excessive workload concentration. Meanwhile, its runtime remains the shortest among all algorithms, increasing only to $3.37\times 10^{-3}$ s at 500 tasks. These results suggest that ASQS maintains efficient scheduling decisions with limited computational overhead.

Taken together, the G2 results indicate that ASQS provides stronger workload robustness under fixed fog-resource capacity. Instead of optimizing only isolated metrics, ASQS maintains a more coherent performance profile in terms of scheduling cost, completion time, latency, load distribution, and decision-making efficiency.

Although the above results demonstrate the performance advantages of ASQS from different scheduling metrics, statistical significance analysis is still required to verify whether these improvements are reliable across repeated independent runs and different fog scheduling scenarios.

#### 5.3.6. Statistical Significance Analysis on Small-Scale Experiments

To check whether the performance differences are statistically reliable, Friedman and Wilcoxon signed-rank tests were conducted based on the fitness results from 30 independent runs. The Friedman test was used to compare all algorithms across the ten fog scheduling scenarios. The Wilcoxon signed-rank test was then used to compare ASQS with each baseline algorithm. Because several pairwise comparisons were performed, the Holm–Bonferroni correction was applied, and the significance level was set to 0.05.

As shown in Table 18, ASQS achieves the best average rank of 1.34 among all ten algorithms. The Friedman statistic is $\chi_{F}^{2}=77.82$ with 9 degrees of freedom, and the corresponding *p*-value is smaller than 0.001. This result shows that the compared algorithms have statistically significant performance differences.

Table 19 reports the corrected pairwise Wilcoxon results. All MFD values are positive, which means that ASQS obtains lower median fitness values than the baseline algorithms. In addition, all corrected *p*-values are below 0.05, indicating that the pairwise differences are statistically significant after multiple-comparison correction. The improvements over EcoTaskSched and NF-MORL are smaller than those over conventional baselines, but ASQS still obtains positive MFD values and more wins than losses.

These results show that ASQS has statistically reliable advantages across both node-scaling and task-scaling fog scheduling scenarios.

#### 5.3.7. Large-Scale Task-Intensive Scalability Analysis

**Figure 11:**
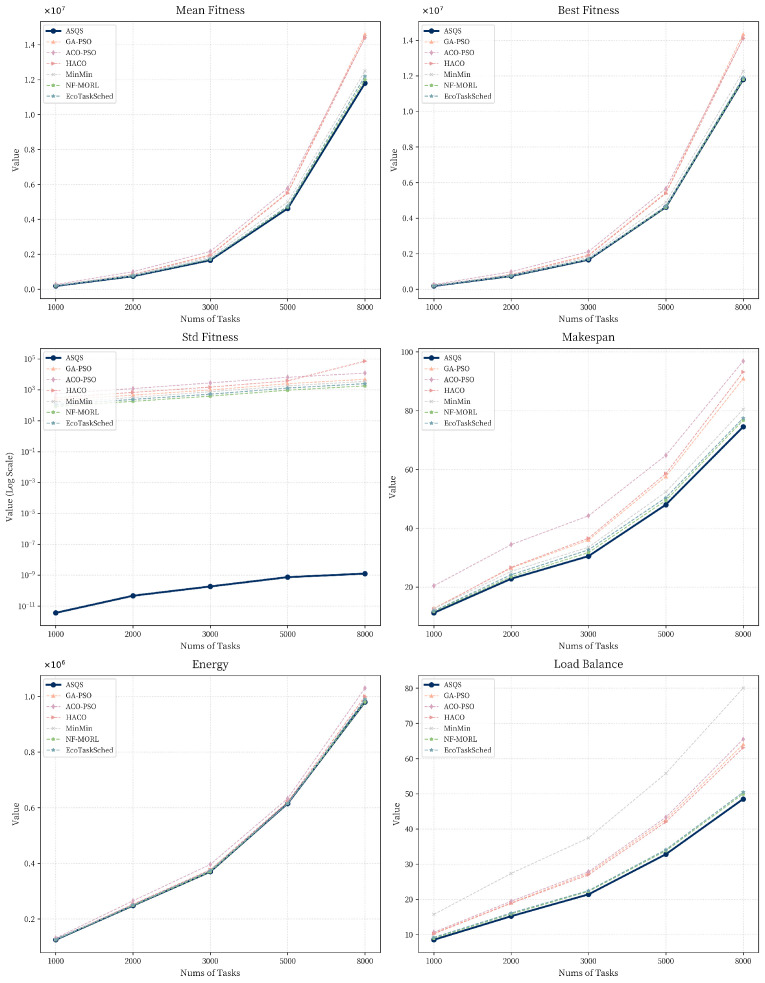
Performance trends of different algorithms under large-scale task-intensive IoT–Fog scheduling scenarios with 100 fog nodes.

Table 20 and Figure 11 report the large-scale task-intensive scheduling results with 100 fog nodes. As the number of IoT tasks increases from 1000 to 8000, all algorithms show higher scheduling costs and longer computational time due to the enlarged search space and heavier workload pressure. Nevertheless, ASQS maintains a stable and competitive performance trend across all scenarios.

ASQS achieves the lowest mean fitness in all five large-scale cases, indicating that its quality-source archive and multi-anchor synchronization mechanism remain effective under high-dimensional scheduling spaces. Moreover, ASQS obtains the best average latency and load-balance results in all scenarios, showing its ability to reduce response delay and avoid excessive workload concentration on a few fog nodes. For makespan, ASQS achieves the best results in most cases, although it does not dominate every scenario. This suggests that ASQS provides a balanced scheduling strategy rather than optimizing only one metric. In addition, ASQS maintains zero constraint violation across all large-scale scenarios, demonstrating strong feasibility preservation under intensive task loads.

Although ASQS requires more runtime than simple deterministic or lightweight methods, its computational cost is comparable to other population-based metaheuristics and is compensated by its superior scheduling quality. Overall, these results confirm that ASQS has good scalability and robustness in large-scale IoT–Fog task scheduling, especially when the task-node ratio increases substantially.

## 6. Conclusions and Future Work

This paper proposed ASQS, a quality-source-driven multi-anchor synchronized search algorithm for IoT–Fog task scheduling. By extracting representative quality-source anchors from layered populations and reusing them through an ACO-inspired probabilistic synchronization mechanism, ASQS improves the utilization of historical high-quality search information and reduces the dependence on a single elite solution.

Extensive experiments, including benchmark-function tests, ablation studies, sensitivity analysis, IoT–Fog scheduling experiments, large-scale task-intensive scenarios, and statistical significance tests, were conducted to evaluate the proposed algorithm. The results show that ASQS achieves competitive optimization accuracy and convergence stability on benchmark functions, while the ablation study verifies the contribution of its key components. In fog scheduling scenarios, ASQS obtains strong comprehensive scheduling performance in terms of fitness, makespan, latency, load balance, and constraint handling. In particular, the large-scale experiment with 100 fog nodes and up to 8000 IoT tasks demonstrates that ASQS can maintain stable performance under increased task dimensions and heavier workload pressure. These findings indicate that ASQS is an effective, robust, and scalable optimizer for resource-constrained IoT–Fog task scheduling.

Despite these results, several limitations remain. The current model focuses on static batch scheduling and does not fully consider dynamic task arrivals, node failures, bandwidth fluctuations, workload changes, or online task migration. In addition, the objective weights and penalty coefficients are predefined, and the algorithm still involves several control parameters. Moreover, although simulation-based experiments provide reproducible evaluation, further validation on real workload traces and fog/edge platforms is required.

Future work will extend ASQS toward dynamic and online computational scheduling in IoT–Fog environments. This includes handling continuously arriving tasks, time-varying resource states, adaptive task migration, and dynamic resource allocation. Adaptive weight adjustment, self-adaptive parameter control, and Pareto-based multi-objective optimization will also be explored to improve flexibility under diverse QoS requirements. Finally, real-world traces and practical fog/edge testbeds will be used to further evaluate the scalability and deployment feasibility of ASQS in realistic dynamic computing environments.

## Figures and Tables

**Table 1:** Summary of the main components in ASQS.

Component	Basis	Function	Effect	Type
Hierarchical organization	HRO-inspired	Quality-layer grouping	Structured diversity	Adapted
FNO-guided search	FNO	Better/worse reference update	Global-local balance	Adapted
Lévy escape perturbation	Lévy flight	Conditional long-jump perturbation	Exploration	Adapted
Quality-source archive	Proposed	Multi-layer elite storage	Multi-center guidance	Proposed
Anchor seeding	Proposed	Anchor-neighborhood generation	Local refinement	Proposed
ACO-inspired synchronization	ACO-inspired	Probabilistic anchor sharing	Cooperative convergence	Hybrid

**Table 2:** Roles of different quality layers in anchor construction.

Layer in ASQS	Fitness-Based Criterion	Function in Anchor Construction
High-quality layer	Top-ranked	Provides exploitation-oriented quality sources.
Medium-quality layer	Middle-ranked	Provides balanced quality sources for maintaining search stability.
Low-quality layer	Bottom-ranked	Provides diversity-oriented sources to enlarge the search scope.

**Table 3:** Unimodal benchmark functions (F01–F08) used to evaluate the exploitation strength of algorithms in smooth landscapes.

Function	Name	Formula	Range	Opt
F01	Shifted Sphere Function	$f_{1}\left(x\right)=\sum_{i=1}^{D}z_{i}^{2}+f_{\mathrm{bias},1},\;z=x-o,\;X=\left[x_{1},x_{2},\ldots ,x_{D}\right],\;o=\left[o_{1},o_{2},\ldots ,o_{D}\right].$	${\left[-100,100\right]}^{D}$	−450
F02	Shifted Schwefel’s Problem 1.2	$f_{2}\left(x\right)=\sum_{i=1}^{D}{\left(\sum_{j=1}^{i}z_{j}\right)}^{2}+f_{\mathrm{bias},2},\;z=x-o,\;X=\left[x_{1},x_{2},\ldots ,x_{D}\right],\;o=\left[o_{1},o_{2},\ldots ,o_{D}\right].$	${\left[-100,100\right]}^{D}$	−450
F03	Shifted Rotated High Conditioned Elliptic Function	$f_{3}\left(x\right)=\sum_{i=1}^{D}{\left(10^{6}\right)}^{\frac{i-1}{D-1}}z_{i}^{2}+f_{\mathrm{bias},3},\;z=\left(x-o\right)M,\;X=\left[x_{1},x_{2},\ldots ,x_{D}\right],\;o=\left[o_{1},o_{2},\ldots ,o_{D}\right].$	${\left[-100,100\right]}^{D}$	−450
F04	Shifted Schwefel’s Problem 1.2 with Noise in Fitness	$f_{4}\left(x\right)=\left[\sum_{i=1}^{D}{\left(\sum_{j=1}^{i}z_{j}\right)}^{2}\right]\left(1+0.4|N(0,1)|\right)+f_{\mathrm{bias},4},\;z=x-o.$	${\left[-100,100\right]}^{D}$	−450
F05	Schwefel’s Problem 2.6 with Global Optimum on Bounds	$f_{5}\left(x\right)=\max_{i}\left\{|A_{i}X-B_{i}|\right\}+f_{\mathrm{bias},5},\;i=1,\ldots ,D,\;X=\left[x_{1},x_{2},\ldots ,x_{D}\right],$ where *A* is a D×D matrix.	${\left[-100,100\right]}^{D}$	−310
F06	High Conditioned Elliptic Function	$f_{6}\left(x\right)=\sum_{i=1}^{D}{\left(10^{6}\right)}^{\frac{i-1}{D-1}}z_{i}^{2}+f_{\mathrm{bias},6},\;z=M\left(x-o\right).$	${\left[-100,100\right]}^{D}$	100
F07	Bent Cigar Function	$f_{7}\left(x\right)=z_{1}^{2}+\sum_{i=2}^{D}\left(10^{6}\right)z_{i}^{2}+f_{\mathrm{bias},7},\;z=M\left(x-o\right).$	${\left[-100,100\right]}^{D}$	200
F08	Discus Function	$f_{8}\left(x\right)=\left(10^{6}\right)z_{1}^{2}+\sum_{i=2}^{D}z_{i}^{2}+f_{\mathrm{bias},8},\;z=M\left(x-o\right).$	${\left[-100,100\right]}^{D}$	300

**Table 4:** Multimodal benchmark functions (F09–F20) assessing exploration performance and the ability to avoid local optima.

Function	Name	Formula	Range	Opt
F09	Rosenbrock	$F09\left(x\right)=\sum_{i=1}^{D-1}\left[100{\left(z_{i}^{2}-z_{i+1}\right)}^{2}+{\left(z_{i}-1\right)}^{2}\right]+f_{\mathrm{bias},9},\;z=M\left(\frac{2.048(x-o)}{100}\right)+1,\;F09=F09\left(z\right).$	${\left[-100,100\right]}^{D}$	400
F10	Ackley	$F10\left(x\right)=20-20\exp\left(-0.2\sqrt{\frac{1}{D}\sum_{i=1}^{D}z_{i}^{2}}\right)-\exp\left(\frac{1}{D}\sum_{i=1}^{D}\cos\left(2\pi z_{i}\right)\right)+e+f_{\mathrm{bias},10},\;z=M\left(x-o\right).$	${\left[-100,100\right]}^{D}$	500
F11	Weierstrass	$F11\left(x\right)=\sum_{i=1}^{D}\left[\sum_{k=0}^{k_{\max}}a^{k}\cos\left(2\pi b^{k}\left(z_{i}+0.5\right)\right)\right]-D\sum_{k=0}^{k_{\max}}a^{k}\cos\left(2\pi b^{k}\cdot 0.5\right)+f_{\mathrm{bias},11},$ a=0.5, b=3, $k_{\max}=20$, $z=M\left(\frac{0.5(x-o)}{100}\right),\;F11=F11\left(z\right).$	${\left[-100,100\right]}^{D}$	600
F12	Griewank	$F12\left(x\right)=\frac{1}{4000}\sum_{i=1}^{D}z_{i}^{2}-\prod_{i=1}^{D}\cos\left(\frac{z_{i}}{\sqrt{i}}\right)+1+f_{\mathrm{bias},12},\;z=M\left(\frac{600(x-o)}{100}\right),\;F12=F12\left(z\right).$	${\left[-100,100\right]}^{D}$	700
F13	Rastrigin	$F13\left(x\right)=\sum_{i=1}^{D}\left[z_{i}^{2}-10\cos\left(2\pi z_{i}\right)+10\right]+f_{\mathrm{bias},13},\;z=M\left(\frac{5.12(x-o)}{100}\right),\;F13=F13\left(z\right).$	${\left[-100,100\right]}^{D}$	800
F14	Modified Schwefel	$F14\left(x\right)=418.9829D-\sum_{i=1}^{D}g\left(q_{i}\right)+f_{\mathrm{bias},14},\;q_{i}=z_{i}+420.9687462275036,\;z=M\left(\frac{1000(x-o)}{100}\right),\;F14=F14\left(z\right).$	${\left[-100,100\right]}^{D}$	900
F15	Katsuura	$F15\left(x\right)=\frac{10}{D^{2}}\prod_{i=1}^{D}{\left[1+i\sum_{j=1}^{32}\frac{\left|2^{j}z_{i}-\mathrm{round}\left(2^{j}z_{i}\right)\right|}{2^{j}}\right]}^{\frac{10}{D^{1.2}}}-\frac{10}{D^{2}}+f_{\mathrm{bias},15},\;z=M\left(\frac{5(x-o)}{100}\right),\;F15=F15\left(z\right).$	${\left[-100,100\right]}^{D}$	1000
F16	HappyCat	$F16\left(x\right)={\left|\sum_{i=1}^{D}z_{i}^{2}-D\right|}^{1/4}+\frac{0.5\sum_{i=1}^{D}z_{i}^{2}+\sum_{i=1}^{D}z_{i}}{D}+0.5+f_{\mathrm{bias},16},\;z=M\left(\frac{5(x-o)}{100}\right),\;F16=F16\left(z\right).$	${\left[-100,100\right]}^{D}$	1100
F17	HGBat	$F17\left(x\right)={\left|{\left(\sum_{i=1}^{D}z_{i}^{2}\right)}^{2}-{\left(\sum_{i=1}^{D}z_{i}\right)}^{2}\right|}^{1/2}+\frac{0.5\sum_{i=1}^{D}z_{i}^{2}+\sum_{i=1}^{D}z_{i}}{D}+0.5+f_{\mathrm{bias},17},\;z=M\left(\frac{5(x-o)}{100}\right),\;F17=F17\left(z\right).$	${\left[-100,100\right]}^{D}$	1200
F18	Expanded Griewank plus Rosenbrock	$F18\left(x\right)=F12\left(f_{9}\left(z_{1},z_{2}\right)\right)+F12\left(f_{9}\left(z_{2},z_{3}\right)\right)+\cdots +F12\left(f_{9}\left(z_{D-1},z_{D}\right)\right)+F12\left(f_{9}\left(z_{D},z_{1}\right)\right)+f_{\mathrm{bias},18},$ $z=M\left(\frac{5(x-o)}{100}\right)+1,\;F18=F18\left(z\right).$	${\left[-100,100\right]}^{D}$	1300
F19	Expanded Scaffer F6	$F19\left(x\right)=g\left(z_{1},z_{2}\right)+g\left(z_{2},z_{3}\right)+\cdots +g\left(z_{D-1},z_{D}\right)+g\left(z_{D},z_{1}\right)+f_{\mathrm{bias},19},$ $g\left(x,y\right)=0.5+\frac{\sin^{2}\left(\sqrt{x^{2}+y^{2}}\right)-0.5}{{\left[1+0.001(x^{2}+y^{2})\right]}^{2}},\;z=M\left(x-o\right)+1,\;F19=F19\left(z\right).$	${\left[-100,100\right]}^{D}$	1400
F20	D-type	$F20\left(x\right)=\left\{\begin{array}{cc} C_{i}\left(x\right), & x\in S_{i},\;i=1,2,\ldots ,m,\\ g(x), & x\in S_{2}\cup \cdots \cup S_{m}, \end{array}\right.$ $C_{i}\left(x\right)=\left(\frac{1}{\beta_{i}}{∥x-M_{i}∥}^{2}-\alpha_{i}\right)∥x-M_{i}{∥}^{2}+f_{i},\;g\left(x\right)={∥x-T∥}^{2}+t,\;m=20.$	${\left[-100,100\right]}^{D}$	1500

**Table 5:** Hybrid benchmark functions (F21–F26) combining multiple subfunctions to test exploration and exploitation performance.

Function	Formulas	Range	Opt
F21 Hybrid Function (HF01)	N=3, F14, F13, F01, p=[0.3,0.3,0.4]	${\left[-100,100\right]}^{D}$	1700
F22 Hybrid Function (HF02)	N=3, f07, F17, F13, p=[0.3,0.3,0.4]	${\left[-100,100\right]}^{D}$	1800
F23 Hybrid Function (HF03)	N=4, F12, F11, f09, F19, p=[0.2,0.2,0.3,0.3]	${\left[-100,100\right]}^{D}$	1900
F24 Hybrid Function (HF04)	N=4, F17, f08, F18, F13, p=[0.2,0.2,0.3,0.3]	${\left[-100,100\right]}^{D}$	2000
F25 Hybrid Function (HF05)	N=5, F19, F17, f09, F14, f06, p=[0.1,0.2,0.2,0.2,0.3]	${\left[-100,100\right]}^{D}$	2100
F26 Hybrid Function (HF06)	N=5, F15, F16, F18, F14, F10, p=[0.1,0.2,0.2,0.2,0.3]	${\left[-100,100\right]}^{D}$	2200

**Table 6:** Composition benchmark functions (F27–F33) incorporating rotated and shifted components.

Function	Parameters	Range	Opt
F27 Composition Function (CF01)	N=5, F09, F06, F07, F08, F06, σ=[10,20,30,40,50] λ=[1,1e−6,1e−26,1e−6,1e−6] bias=[0,100,200,300,400]	${\left[-100,100\right]}^{D}$	2300
F28 Composition Function (CF02)	N=3, F10, F09, F11, σ=[20,20,20] λ=[1,1,1] bias=[0,100,200]	${\left[-100,100\right]}^{D}$	2400
F29 Composition Function (CF03)	N=3, F11, F09, F01, σ=[10,30,50] λ=[0.25,1,1e−7] bias=[0,100,200]	${\left[-100,100\right]}^{D}$	2500
F30 Composition Function (CF04)	N=5, F11, F13, F01, F06, F07, σ=[10,10,10,10,10] λ=[0.25,1,1e−7,2.5,10] bias=[0,100,200,300,400]	${\left[-100,100\right]}^{D}$	2600
F31 Composition Function (CF05)	N=5, F14, F09, F11, F06, F01, σ=[10,10,10,10,10] λ=[10,10,2.5,25,1e−6] bias=[0,100,200,300,400]	${\left[-100,100\right]}^{D}$	2700
F32 Composition Function (CF06)	N=5, F15, F13, F11, F16, F01, σ=[10,20,30,40,50] λ=[2.5,10,2.5,5e−4,1e−6] bias=[0,100,200,300,400]	${\left[-100,100\right]}^{D}$	2800
F33 Composition Function (CF07)	N=3, F17, F18, F19, σ=[10,30,50] λ=[1,1,1] bias=[0,100,200]	${\left[-100,100\right]}^{D}$	2900

**Table 7:** Setting of algorithm parameters.

Algorithms	Parameters
All	dim=30,nPop=30,numRuns=30
ASQS	$numPopulations=3,\;w=0.9-0.5\times ratio,\;c_{1}=c_{2}=c_{3}=1.5,$ β=1.5,λ=0.01,τ=0.1×searchRange×(1−ratio), $s_{\tau }=\left[0.6,1.0,1.4\right],\;s_{a}=\left[0.2,0.3,0.4\right],\;V_{0}=0$
CMA	$numPopulations=3,\;V_{0}=0,$ $V_{\max}=1,\;w_{\max}=0.9,\;w_{\min}=0.2,\;C_{1}=2,\;C_{2}=2,\;\alpha_{CMA}=2,$ $p=0.6,\;numAnts=50,\;\alpha_{ACO}=1,\;\beta_{ACO}=2,\;\rho =0.1,\;Q=1$
ACO	$q=0.5,\;\chi_{i}=0.8$
FNO	$\alpha =\sqrt{1-\frac{FEs}{MaxFEs}},\;\phi \in \left\{0,1\right\},\;r_{1}\in \left[0,1\right],\;\mu \in \left[0,1\right],$ DFF=|μ−α+2α·rand|,W=(2+U(−1,1))×U(1,2)
MetaDE	F=0.5,CR=0.9
HSA	HMCR=0.995,PAR=0.9,bw=0.01
DE	F=0.5,CR=0.9
GWO	a=linear-decrease-from-2-to-0,C=random[0,2]
HPSOBOA	$w_{\max}=0.9,\;w_{\min}=0.4,\;c_{1}=2.0,\;c_{2}=0.2,\;vMaxRatio=0.1$ $a=0.1,\;c_{0}=0.01,\;pBOA_{\max}=0.8,\;pBOA_{\min}=0.2$
PS-BES	$w_{\max}=0.9,\;w_{\min}=0.4,\;c_{1}=2.0,\;c_{2}=2.0,\;vMaxRatio=0.1,$ $a=10,\;R=1.5,\;\beta_{\max}=0.9,\;\beta_{\min}=0.1,\;K=20,\;q=0.2$
PSOBOA	$w_{\max}=0.9,\;w_{\min}=0.4,\;c_{1\max}=2.5,\;c_{1\min}=0.5$ $c_{2\max}=2.5,\;c_{2\min}=0.5,\;vMaxRatio=0.1,\;a=0.1,\;c_{0}=0.01$ $pBOA_{\max}=0.8,\;pBOA_{\min}=0.2,\;\beta_{\max}=0.9,\;\beta_{\min}=0.1$
WOA	b=1

**Table 8:** Comparison results of all algorithms on 33 benchmark functions.

Func.	Metrics	ASQS	CMA	FNO	PSO	ACO	HSA	GWO	WOA	DE	MetaDE	HPSOBOA	PS-BES	PSOBOA
F1	Avg	0	$1.2039\times 10^{-1}$	0	$3.3333\times 10^{2}$	0	0	$2.3701\times 10^{-2}$	0	4.0027	0	0	$2.3117\times 10^{-6}$	0
	Std	0	$3.7468\times 10^{-2}$	0	$1.8257\times 10^{3}$	0	0	$6.0355\times 10^{-3}$	0	$1.3815\times 10^{1}$	0	0	$1.0979\times 10^{-5}$	0
	Rank	4.50	11	4.50	13	4.50	4.50	10	4.50	12	4.50	4.50	9	4.50
F2	Avg	0	0	0	$5.168\times 10^{4}$	0	$1.7253\times 10^{5}$	$5.0968\times 10^{3}$	$5.5282\times 10^{2}$	0	0	$2.781\times 10^{2}$	0	0
	Std	$7.3379\times 10^{-1}$	5.0289	$1.4165\times 10^{3}$	$1.9279\times 10^{4}$	$2.1992\times 10^{-6}$	$6.1266\times 10^{4}$	$2.3742\times 10^{3}$	$9.3086\times 10^{2}$	$1.0717\times 10^{2}$	$2.7527\times 10^{2}$	$5.9221\times 10^{2}$	$1.1146\times 10^{-1}$	$6.0288\times 10^{2}$
	Rank	4.50	4.50	4.50	12	4.50	13	11	10	4.50	4.50	9	4.50	4.50
F3	Avg	$6.0123\times 10^{5}$	$4.1485\times 10^{6}$	$3.5658\times 10^{6}$	$1.7317\times 10^{8}$	$2.0531\times 10^{6}$	$1.5456\times 10^{9}$	$1.1892\times 10^{7}$	$7.602\times 10^{6}$	$2.1604\times 10^{6}$	$3.4903\times 10^{6}$	$6.0806\times 10^{6}$	$1.1707\times 10^{6}$	$7.1385\times 10^{6}$
	Std	$2.6996\times 10^{5}$	$1.5974\times 10^{6}$	$2.1901\times 10^{6}$	$1.0517\times 10^{8}$	$1.4705\times 10^{6}$	$4.4747\times 10^{8}$	$7.9495\times 10^{6}$	$4.0255\times 10^{6}$	$7.7296\times 10^{5}$	$1.5455\times 10^{6}$	$4.293\times 10^{6}$	$6.2524\times 10^{5}$	$4.5718\times 10^{6}$
	Rank	1	7	6	12	3	13	11	10	4	5	8	2	9
F4	Avg	0	0	$4.5244\times 10^{3}$	$5.3418\times 10^{4}$	0	$1.377\times 10^{5}$	$1.338\times 10^{4}$	$7.0718\times 10^{3}$	0	$1.1237\times 10^{4}$	$7.9076\times 10^{3}$	0	$1.0378\times 10^{4}$
	Std	8.5930	$2.2691\times 10^{2}$	$5.1118\times 10^{3}$	$2.5874\times 10^{4}$	$1.6723\times 10^{2}$	$2.5766\times 10^{4}$	$3.8256\times 10^{3}$	$3.9819\times 10^{3}$	$3.6307\times 10^{1}$	$4.2904\times 10^{3}$	$2.9408\times 10^{3}$	$2.5974\times 10^{1}$	$4.3495\times 10^{3}$
	Rank	3	3	6	12	3	13	11	7	3	10	8	3	9
F5	Avg	0	0	$3.013\times 10^{3}$	$2.5506\times 10^{4}$	0	$6.0562\times 10^{4}$	$1.3024\times 10^{4}$	$2.5033\times 10^{3}$	0	$2.682\times 10^{4}$	$5.1778\times 10^{3}$	$3.1605\times 10^{1}$	$3.6956\times 10^{3}$
	Std	0	$5.1506\times 10^{1}$	$6.5065\times 10^{3}$	$7.5231\times 10^{3}$	$3.9354\times 10^{2}$	$5.3321\times 10^{3}$	$2.5758\times 10^{3}$	$4.6823\times 10^{3}$	$2.1002\times 10^{2}$	$3.3409\times 10^{3}$	$7.738\times 10^{3}$	$1.871\times 10^{3}$	$6.6426\times 10^{3}$
	Rank	2.50	2.50	7	11	2.50	13	10	6	2.50	12	9	5	8
F6	Avg	$1.9999\times 10^{6}$	$3.5645\times 10^{6}$	$2.4351\times 10^{6}$	$1.5322\times 10^{8}$	$1.3775\times 10^{6}$	$1.3597\times 10^{9}$	$1.1873\times 10^{7}$	$6.7835\times 10^{6}$	$3.9723\times 10^{5}$	$2.8243\times 10^{6}$	$4.7227\times 10^{6}$	$1.061\times 10^{6}$	$6.9227\times 10^{6}$
	Std	$8.308\times 10^{5}$	$1.3346\times 10^{6}$	$1.6634\times 10^{6}$	$1.0738\times 10^{8}$	$5.8209\times 10^{5}$	$1.7972\times 10^{8}$	$6.083\times 10^{6}$	$2.9872\times 10^{6}$	$2.3962\times 10^{5}$	$1.5815\times 10^{6}$	$2.0751\times 10^{6}$	$7.8865\times 10^{5}$	$3.6604\times 10^{6}$
	Rank	4	7	5	12	3	13	11	9	1	6	8	2	10
F7	Avg	$5.9358\times 10^{4}$	$1.7202\times 10^{5}$	$1.5835\times 10^{8}$	$1.6433\times 10^{10}$	$9.855\times 10^{3}$	$4.8267\times 10^{10}$	$4.8139\times 10^{8}$	$1.888\times 10^{7}$	$6.381\times 10^{6}$	$3.2582\times 10^{7}$	$5.4276\times 10^{6}$	$8.0163\times 10^{3}$	$1.8155\times 10^{5}$
	Std	$1.5863\times 10^{5}$	$4.2027\times 10^{5}$	$5.5176\times 10^{8}$	$6.6448\times 10^{9}$	$9.2354\times 10^{3}$	$4.7739\times 10^{9}$	$4.575\times 10^{8}$	$4.2632\times 10^{7}$	$3.3836\times 10^{7}$	$1.2284\times 10^{8}$	$2.2521\times 10^{7}$	$6.1161\times 10^{3}$	$6.7852\times 10^{5}$
	Rank	3	4	10	12	2	13	11	8	7	9	6	1	5
F8	Avg	$5.497\times 10^{2}$	$1.191\times 10^{3}$	$2.0798\times 10^{4}$	$8.1726\times 10^{4}$	$6.8999\times 10^{3}$	$6.5266\times 10^{4}$	$2.57\times 10^{4}$	$7.2127\times 10^{3}$	$1.4675\times 10^{3}$	$1.7439\times 10^{4}$	$5.3129\times 10^{3}$	$3.447\times 10^{2}$	$5.4466\times 10^{3}$
	Std	$9.4811\times 10^{1}$	$3.6252\times 10^{2}$	$5.5662\times 10^{3}$	$3.7877\times 10^{4}$	$2.6462\times 10^{3}$	$7.2796\times 10^{3}$	$6.4067\times 10^{3}$	$3.3406\times 10^{3}$	$9.9065\times 10^{2}$	$5.1864\times 10^{3}$	$1.3321\times 10^{3}$	$2.0569\times 10^{1}$	$1.8068\times 10^{3}$
	Rank	2	3	10	13	7	12	11	8	4	9	5	1	6
F9	Avg	$4.29\times 10^{2}$	$4.6892\times 10^{2}$	$4.9658\times 10^{2}$	$2.8682\times 10^{3}$	$4.6147\times 10^{2}$	$1.7997\times 10^{4}$	$6.4439\times 10^{2}$	$5.0343\times 10^{2}$	$4.6175\times 10^{2}$	$4.907\times 10^{2}$	$4.8847\times 10^{2}$	$4.6538\times 10^{2}$	$4.4735\times 10^{2}$
	Std	$3.295\times 10^{1}$	$1.9442\times 10^{1}$	$5.9847\times 10^{1}$	$1.1939\times 10^{3}$	$3.0625\times 10^{1}$	$2.9844\times 10^{3}$	$7.7666\times 10^{1}$	$3.7986\times 10^{1}$	$4.7303\times 10^{1}$	$5.4244\times 10^{1}$	$3.1503\times 10^{1}$	$2.3803\times 10^{1}$	$3.7811\times 10^{1}$
	Rank	1	6	9	12	3	13	11	10	4	8	7	5	2
F10	Avg	$5.2095\times 10^{2}$	$5.2026\times 10^{2}$	$5.2097\times 10^{2}$	$5.2088\times 10^{2}$	$5.2014\times 10^{2}$	$5.2101\times 10^{2}$	$5.2094\times 10^{2}$	$5.2026\times 10^{2}$	$5.2096\times 10^{2}$	$5.2003\times 10^{2}$	$5.2095\times 10^{2}$	$5.2093\times 10^{2}$	$5.2093\times 10^{2}$
	Std	$4.3691\times 10^{-2}$	$9.0695\times 10^{-2}$	$6.046\times 10^{-2}$	$1.0309\times 10^{-1}$	$9.938\times 10^{-2}$	$7.4294\times 10^{-2}$	$5.5456\times 10^{-2}$	$1.1776\times 10^{-1}$	$3.6591\times 10^{-2}$	$6.4637\times 10^{-2}$	$5.0587\times 10^{-2}$	$5.6894\times 10^{-2}$	$4.7343\times 10^{-2}$
	Rank	9	3	12	5	2	13	8	4	11	1	10	6	7
F11	Avg	$6.0815\times 10^{2}$	$6.1128\times 10^{2}$	$6.2312\times 10^{2}$	$6.2946\times 10^{2}$	$6.0992\times 10^{2}$	$6.4305\times 10^{2}$	$6.1383\times 10^{2}$	$6.2615\times 10^{2}$	$6.1027\times 10^{2}$	$6.3464\times 10^{2}$	$6.2912\times 10^{2}$	$6.1342\times 10^{2}$	$6.3056\times 10^{2}$
	Std	2.3135	3.1567	4.9851	3.1500	4.1745	1.1983	2.6097	3.0299	3.3578	2.9218	3.2554	4.0636	3.9144
	Rank	1	4	7	10	2	13	6	8	3	12	9	5	11
F12	Avg	$7\times 10^{2}$	$7.003\times 10^{2}$	$7.0705\times 10^{2}$	$9.112\times 10^{2}$	$7.0009\times 10^{2}$	$1.2404\times 10^{3}$	$7.1597\times 10^{2}$	$7.0114\times 10^{2}$	$7.0066\times 10^{2}$	$7.0001\times 10^{2}$	$7.0001\times 10^{2}$	$7.0001\times 10^{2}$	$7.0001\times 10^{2}$
	Std	$6.1646\times 10^{-3}$	$7.9615\times 10^{-2}$	$1.012\times 10^{1}$	$7.3081\times 10^{1}$	$3.7641\times 10^{-2}$	$4.5547\times 10^{1}$	$1.7796\times 10^{1}$	$2.7353\times 10^{-1}$	2.1607	$2.106\times 10^{-2}$	$1.1604\times 10^{-2}$	$1.0967\times 10^{-2}$	$1.7134\times 10^{-2}$
	Rank	1	7	10	12	6	13	11	9	8	4	3	2	5
F13	Avg	$9.716\times 10^{2}$	$8.7919\times 10^{2}$	$8.7589\times 10^{2}$	$1.0078\times 10^{3}$	$8.5097\times 10^{2}$	$1.1587\times 10^{3}$	$9.1129\times 10^{2}$	$9.5922\times 10^{2}$	$8.3806\times 10^{2}$	$9.7584\times 10^{2}$	$9.7108\times 10^{2}$	$9.0099\times 10^{2}$	$9.5826\times 10^{2}$
	Std	$1.02\times 10^{1}$	$1.9561\times 10^{1}$	$2.7833\times 10^{1}$	$4.7602\times 10^{1}$	$1.302\times 10^{1}$	$1.9977\times 10^{1}$	$5.029\times 10^{1}$	$3.6951\times 10^{1}$	$1.8907\times 10^{1}$	$3.0226\times 10^{1}$	$5.834\times 10^{1}$	$2.6625\times 10^{1}$	$3.3175\times 10^{1}$
	Rank	10	4	3	12	2	13	6	8	1	11	9	5	7
F14	Avg	$7.34\times 10^{3}$	$4.0112\times 10^{3}$	$7.5278\times 10^{3}$	$6.1461\times 10^{3}$	$3.5217\times 10^{3}$	$9.4984\times 10^{3}$	$4.1743\times 10^{3}$	$5.0993\times 10^{3}$	$6.6929\times 10^{3}$	$5.557\times 10^{3}$	$7.1113\times 10^{3}$	$4.0558\times 10^{3}$	$5.9973\times 10^{3}$
	Std	$2.7351\times 10^{2}$	$6.7415\times 10^{2}$	$7.1767\times 10^{2}$	$6.9473\times 10^{2}$	$6.3139\times 10^{2}$	$4.299\times 10^{2}$	$1.1708\times 10^{3}$	$7.4809\times 10^{2}$	$1.2339\times 10^{3}$	$6.5148\times 10^{2}$	$4.0515\times 10^{2}$	$6.6757\times 10^{2}$	$7.9618\times 10^{2}$
	Rank	11	2	12	8	1	13	4	5	9	6	10	3	7
F15	Avg	$1.0025\times 10^{3}$	$1.0005\times 10^{3}$	$1.0027\times 10^{3}$	$1.0016\times 10^{3}$	$1.0002\times 10^{3}$	$1.0035\times 10^{3}$	$1.0025\times 10^{3}$	$1.0008\times 10^{3}$	$1.0024\times 10^{3}$	$1.0017\times 10^{3}$	$1.0024\times 10^{3}$	$1.0017\times 10^{3}$	$1.0022\times 10^{3}$
	Std	$2.4656\times 10^{-1}$	$3.4331\times 10^{-1}$	$2.8183\times 10^{-1}$	$4.589\times 10^{-1}$	$8.1847\times 10^{-2}$	$5.1174\times 10^{-1}$	$2.3915\times 10^{-1}$	$3.3747\times 10^{-1}$	$3.1214\times 10^{-1}$	$5.2577\times 10^{-1}$	$3.4201\times 10^{-1}$	$7.4085\times 10^{-1}$	$3.3492\times 10^{-1}$
	Rank	10	2	12	4	1	13	11	3	8	5	9	6	7
F16	Avg	$1.1003\times 10^{3}$	$1.1005\times 10^{3}$	$1.1005\times 10^{3}$	$1.1034\times 10^{3}$	$1.1003\times 10^{3}$	$1.1054\times 10^{3}$	$1.1004\times 10^{3}$	$1.1005\times 10^{3}$	$1.1003\times 10^{3}$	$1.1008\times 10^{3}$	$1.1008\times 10^{3}$	$1.1003\times 10^{3}$	$1.1007\times 10^{3}$
	Std	$5.6853\times 10^{-2}$	$1.3206\times 10^{-1}$	$1.1585\times 10^{-1}$	$6.0123\times 10^{-1}$	$6.0565\times 10^{-2}$	$2.4728\times 10^{-1}$	$8.0801\times 10^{-2}$	$1.0129\times 10^{-1}$	$5.724\times 10^{-2}$	$1.6566\times 10^{-1}$	$1.6275\times 10^{-1}$	$7.3257\times 10^{-2}$	$1.4373\times 10^{-1}$
	Rank	2	7	6	12	3	13	5	8	1	11	10	4	9
F17	Avg	$1.2004\times 10^{3}$	$1.2005\times 10^{3}$	$1.2006\times 10^{3}$	$1.2468\times 10^{3}$	$1.2004\times 10^{3}$	$1.3202\times 10^{3}$	$1.2009\times 10^{3}$	$1.2005\times 10^{3}$	$1.2004\times 10^{3}$	$1.2009\times 10^{3}$	$1.2008\times 10^{3}$	$1.2005\times 10^{3}$	$1.2009\times 10^{3}$
	Std	$1.7065\times 10^{-1}$	$3.5845\times 10^{-1}$	$2.3635\times 10^{-1}$	$1.4293\times 10^{1}$	$2.7026\times 10^{-1}$	8.8024	1.9019	$2.838\times 10^{-1}$	$1.6754\times 10^{-1}$	$3.7228\times 10^{-1}$	$4.1125\times 10^{-1}$	$2.4011\times 10^{-1}$	$3.5008\times 10^{-1}$
	Rank	1	6	7	12	2	13	11	4	3	10	8	5	9
F18	Avg	$1.315\times 10^{3}$	$1.3099\times 10^{3}$	$1.3179\times 10^{3}$	$6.7988\times 10^{4}$	$1.3056\times 10^{3}$	$1.5269\times 10^{6}$	$1.3427\times 10^{3}$	$1.3125\times 10^{3}$	$1.3039\times 10^{3}$	$2.65\times 10^{3}$	$1.3331\times 10^{3}$	$1.3067\times 10^{3}$	$1.3359\times 10^{3}$
	Std	$9.7241\times 10^{-1}$	2.3843	$1.5138\times 10^{1}$	$1.0206\times 10^{5}$	1.8242	$7.595\times 10^{5}$	$4.2162\times 10^{1}$	3.2944	2.3394	$1.1425\times 10^{3}$	$1.215\times 10^{1}$	3.6503	$1.4277\times 10^{1}$
	Rank	6	4	7	12	2	13	10	5	1	11	8	3	9
F19	Avg	$1.4121\times 10^{3}$	$1.4112\times 10^{3}$	$1.4124\times 10^{3}$	$1.4117\times 10^{3}$	$1.411\times 10^{3}$	$1.4132\times 10^{3}$	$1.4109\times 10^{3}$	$1.4119\times 10^{3}$	$1.4116\times 10^{3}$	$1.4125\times 10^{3}$	$1.4115\times 10^{3}$	$1.411\times 10^{3}$	$1.412\times 10^{3}$
	Std	$3.5568\times 10^{-1}$	$5.8404\times 10^{-1}$	$5.1671\times 10^{-1}$	$4.7251\times 10^{-1}$	$6.7601\times 10^{-1}$	$2.5503\times 10^{-1}$	$6.9625\times 10^{-1}$	$5.4209\times 10^{-1}$	$4.7753\times 10^{-1}$	$5.8201\times 10^{-1}$	$3.7422\times 10^{-1}$	$5.9026\times 10^{-1}$	$4.6359\times 10^{-1}$
	Rank	10	4	11	7	2	13	1	8	6	12	5	3	9
F20	Avg	$1.51\times 10^{3}$	$1.5101\times 10^{3}$	$1.5982\times 10^{3}$	$2.181\times 10^{4}$	$1.51\times 10^{3}$	$4.888\times 10^{4}$	$2.5483\times 10^{3}$	$1.5207\times 10^{3}$	$1.5443\times 10^{3}$	$1.51\times 10^{3}$	$1.51\times 10^{3}$	$1.51\times 10^{3}$	$1.51\times 10^{3}$
	Std	0	$3.3133\times 10^{-2}$	$3.3552\times 10^{2}$	$6.925\times 10^{3}$	$4.9286\times 10^{-3}$	$3.3337\times 10^{3}$	$9.698\times 10^{2}$	$2.1482\times 10^{1}$	$9.8496\times 10^{1}$	0	0	$4.008\times 10^{-6}$	0
	Rank	1	7	10	12	6	13	11	8	9	3	4	5	2
F21	Avg	$2.0047\times 10^{3}$	$2.5735\times 10^{3}$	$2.6329\times 10^{3}$	$6.0958\times 10^{3}$	$2.3942\times 10^{3}$	$1.7163\times 10^{4}$	$2.6549\times 10^{3}$	$2.9779\times 10^{3}$	$2.1362\times 10^{3}$	$3.1279\times 10^{3}$	$2.8424\times 10^{3}$	$2.4654\times 10^{3}$	$2.9231\times 10^{3}$
	Std	$2.8499\times 10^{2}$	$2.8023\times 10^{2}$	$2.9101\times 10^{2}$	$1.6423\times 10^{3}$	$2.2014\times 10^{2}$	$2.1706\times 10^{3}$	$2.2677\times 10^{2}$	$3.5691\times 10^{2}$	$2.2472\times 10^{2}$	$5.1797\times 10^{2}$	$3.1638\times 10^{2}$	$2.5886\times 10^{2}$	$3.463\times 10^{2}$
	Rank	1	5	6	12	3	13	7	10	2	11	8	4	9
F22	Avg	$8.8627\times 10^{3}$	$1.1051\times 10^{4}$	$1.4295\times 10^{4}$	$3.6477\times 10^{8}$	$1.1446\times 10^{4}$	$5.4531\times 10^{9}$	$5.3782\times 10^{6}$	$1.3554\times 10^{4}$	$5.9887\times 10^{3}$	$1.3809\times 10^{4}$	$1.1365\times 10^{4}$	$1.1152\times 10^{4}$	$1.099\times 10^{4}$
	Std	$6.112\times 10^{3}$	$7.6653\times 10^{3}$	$7.967\times 10^{3}$	$4.6546\times 10^{8}$	$8.2488\times 10^{3}$	$1.7496\times 10^{9}$	$1.4012\times 10^{7}$	$7.7751\times 10^{3}$	$4.6955\times 10^{3}$	$7.5042\times 10^{3}$	$7.6607\times 10^{3}$	$7.0904\times 10^{3}$	$7.0068\times 10^{3}$
	Rank	2	4	10	12	7	13	11	8	1	9	6	5	3
F23	Avg	$1.9123\times 10^{3}$	$1.9143\times 10^{3}$	$1.9448\times 10^{3}$	$2.1313\times 10^{3}$	$1.9088\times 10^{3}$	$4.8009\times 10^{3}$	$1.92\times 10^{3}$	$1.9167\times 10^{3}$	$1.9082\times 10^{3}$	$1.9479\times 10^{3}$	$1.9131\times 10^{3}$	$1.9169\times 10^{3}$	$1.9159\times 10^{3}$
	Std	$1.0265\times 10^{1}$	3.6519	$2.52\times 10^{1}$	$1.3584\times 10^{2}$	2.8043	$1.032\times 10^{3}$	$1.1784\times 10^{1}$	9.7608	3.9897	$2.2983\times 10^{1}$	2.6099	$1.1348\times 10^{1}$	3.7461
	Rank	3	5	10	12	2	13	9	7	1	11	4	8	6
F24	Avg	$2.2558\times 10^{3}$	$2.3186\times 10^{3}$	$6.6671\times 10^{3}$	$3.8518\times 10^{4}$	$2.2475\times 10^{3}$	$7.8681\times 10^{5}$	$9.1881\times 10^{3}$	$2.4399\times 10^{3}$	$3.7794\times 10^{3}$	$5.7776\times 10^{3}$	$2.607\times 10^{3}$	$2.1295\times 10^{3}$	$2.7177\times 10^{3}$
	Std	$9.7911\times 10^{1}$	$5.9007\times 10^{1}$	$5.6156\times 10^{3}$	$3.0258\times 10^{4}$	$5.4503\times 10^{1}$	$9.7339\times 10^{5}$	$4.3128\times 10^{3}$	$4.0069\times 10^{2}$	$2.4166\times 10^{3}$	$2.3296\times 10^{3}$	$5.1817\times 10^{2}$	$3.7408\times 10^{1}$	$4.9144\times 10^{2}$
	Rank	3	4	10	12	2	13	11	5	8	9	6	1	7
F25	Avg	$2.7979\times 10^{4}$	$1.4143\times 10^{5}$	$4.5142\times 10^{5}$	$1.8721\times 10^{6}$	$4.3482\times 10^{4}$	$5.9983\times 10^{7}$	$5.1571\times 10^{5}$	$2.5782\times 10^{5}$	$3.0077\times 10^{4}$	$1.0017\times 10^{5}$	$1.4365\times 10^{5}$	$2.0503\times 10^{4}$	$1.474\times 10^{5}$
	Std	$2.0526\times 10^{4}$	$1.0239\times 10^{5}$	$1.2316\times 10^{6}$	$2.2516\times 10^{6}$	$2.5696\times 10^{4}$	$6.0468\times 10^{7}$	$6.4685\times 10^{5}$	$2.454\times 10^{5}$	$2.6471\times 10^{4}$	$1.1999\times 10^{5}$	$9.6712\times 10^{4}$	$1.3767\times 10^{4}$	$1.1376\times 10^{5}$
	Rank	2	6	10	12	4	13	11	9	3	5	7	1	8
F26	Avg	$2.3873\times 10^{3}$	$2.501\times 10^{3}$	$2.467\times 10^{3}$	$2.9798\times 10^{3}$	$2.4717\times 10^{3}$	$3.3345\times 10^{4}$	$2.505\times 10^{3}$	$2.591\times 10^{3}$	$2.3795\times 10^{3}$	$3.0678\times 10^{3}$	$2.5918\times 10^{3}$	$2.4683\times 10^{3}$	$2.7321\times 10^{3}$
	Std	$1.8723\times 10^{2}$	$1.4894\times 10^{2}$	$1.394\times 10^{2}$	$4.036\times 10^{2}$	$1.4517\times 10^{2}$	$3.537\times 10^{4}$	$1.3879\times 10^{2}$	$1.7758\times 10^{2}$	$1.5544\times 10^{2}$	$3.0451\times 10^{2}$	$1.8375\times 10^{2}$	$1.2693\times 10^{2}$	$2.1606\times 10^{2}$
	Rank	2	6	3	11	5	13	7	8	1	12	9	4	10
F27	Avg	$9.1201\times 10^{6}$	$2.945\times 10^{7}$	$1.1427\times 10^{7}$	$3.1931\times 10^{8}$	$2.3146\times 10^{7}$	$9.901\times 10^{9}$	$5.0636\times 10^{7}$	$2.6758\times 10^{7}$	$7.0888\times 10^{6}$	$3.3762\times 10^{7}$	$8.4085\times 10^{7}$	$2.1434\times 10^{7}$	$4.9171\times 10^{7}$
	Std	$9.0034\times 10^{6}$	$2.3591\times 10^{7}$	$1.2121\times 10^{7}$	$3.1849\times 10^{8}$	$1.8906\times 10^{7}$	$3.5908\times 10^{9}$	$5.3747\times 10^{7}$	$2.9469\times 10^{7}$	$1.2699\times 10^{6}$	$3.7577\times 10^{7}$	$7.1404\times 10^{7}$	$1.9748\times 10^{7}$	$3.36\times 10^{7}$
	Rank	2	7	3	12	5	13	10	6	1	8	11	4	9
F28	Avg	$2.5445\times 10^{3}$	$2.5433\times 10^{3}$	$2.5603\times 10^{3}$	$2.8122\times 10^{3}$	$2.5411\times 10^{3}$	$4.031\times 10^{3}$	$2.5582\times 10^{3}$	$2.5499\times 10^{3}$	$2.5484\times 10^{3}$	$2.5956\times 10^{3}$	$2.5542\times 10^{3}$	$2.5441\times 10^{3}$	$2.5566\times 10^{3}$
	Std	2.3690	2.0734	$1.4929\times 10^{1}$	$1.124\times 10^{2}$	1.3056	$3.85\times 10^{2}$	$1.895\times 10^{1}$	3.8567	3.4164	$4.8124\times 10^{1}$	5.7675	2.3990	7.3250
	Rank	4	2	10	12	1	13	9	6	5	11	7	3	8
F29	Avg	$4.4778\times 10^{3}$	$4.357\times 10^{3}$	$4.7083\times 10^{3}$	$1.4095\times 10^{4}$	$4.3114\times 10^{3}$	$3.9789\times 10^{5}$	$5.5609\times 10^{3}$	$4.4212\times 10^{3}$	$4.4792\times 10^{3}$	$6.2327\times 10^{3}$	$4.9548\times 10^{3}$	$3.3578\times 10^{3}$	$4.8355\times 10^{3}$
	Std	5.0687	$5.6036\times 10^{2}$	$6.4664\times 10^{2}$	$8.7612\times 10^{3}$	$5.4489\times 10^{2}$	$1.0914\times 10^{5}$	$1.7331\times 10^{3}$	$3.233\times 10^{2}$	9.8259	$1.8347\times 10^{3}$	$3.0359\times 10^{3}$	$8.714\times 10^{2}$	$2.5843\times 10^{3}$
	Rank	5	3	7	12	2	13	10	4	6	11	9	1	8
F30	Avg	$2.628\times 10^{3}$	$2.7655\times 10^{6}$	$1.8797\times 10^{6}$	$8.9656\times 10^{6}$	$6.6606\times 10^{5}$	$4.1932\times 10^{11}$	$1.8077\times 10^{6}$	$1.2965\times 10^{5}$	$2.6331\times 10^{3}$	$3.0582\times 10^{6}$	$1.2073\times 10^{7}$	$2.111\times 10^{6}$	$7.3381\times 10^{6}$
	Std	$9.6132\times 10^{1}$	$6.8636\times 10^{6}$	$1.0281\times 10^{7}$	$3.4464\times 10^{7}$	$3.6329\times 10^{6}$	$7.7762\times 10^{11}$	$6.962\times 10^{6}$	$6.9558\times 10^{5}$	$1.0066\times 10^{2}$	$6.8961\times 10^{6}$	$1.2539\times 10^{7}$	$6.0494\times 10^{6}$	$9.4444\times 10^{6}$
	Rank	1	8	6	11	4	13	5	3	2	9	12	7	10
F31	Avg	$2.877\times 10^{3}$	$3.9255\times 10^{3}$	$3.5971\times 10^{3}$	$1.1694\times 10^{5}$	$5.0365\times 10^{3}$	$2.328\times 10^{12}$	$4.5653\times 10^{3}$	$4.3982\times 10^{3}$	$3.5929\times 10^{3}$	$6.6224\times 10^{3}$	$2.125\times 10^{4}$	$6.4155\times 10^{3}$	$9.6264\times 10^{3}$
	Std	$1.1341\times 10^{1}$	$4.8587\times 10^{3}$	$3.7907\times 10^{3}$	$5.9042\times 10^{5}$	$5.6009\times 10^{3}$	$5.0142\times 10^{12}$	$9.1833\times 10^{3}$	$6.3249\times 10^{3}$	$3.0883\times 10^{3}$	$1.0849\times 10^{4}$	$7.4479\times 10^{4}$	$8.914\times 10^{3}$	$1.5118\times 10^{4}$
	Rank	1	4	3	12	7	13	6	5	2	9	11	8	10
F32	Avg	$3.8039\times 10^{6}$	$7.0678\times 10^{10}$	$5.1254\times 10^{13}$	$6.8821\times 10^{15}$	$1.4468\times 10^{10}$	$1.876\times 10^{16}$	$1.0729\times 10^{15}$	$1.13\times 10^{13}$	$7.8908\times 10^{11}$	$3.8039\times 10^{6}$	$3.8069\times 10^{6}$	$3.9691\times 10^{6}$	$3.8039\times 10^{6}$
	Std	0	$1.7352\times 10^{10}$	$1.9505\times 10^{14}$	$2.7408\times 10^{15}$	$3.0843\times 10^{9}$	$1.5401\times 10^{15}$	$1.0684\times 10^{15}$	$1.7564\times 10^{13}$	$1.6777\times 10^{12}$	$8.4747\times 10^{1}$	$1.0086\times 10^{4}$	$8.0856\times 10^{5}$	$1.8937\times 10^{-8}$
	Rank	1	7	10	12	6	13	11	9	8	3	4	5	2
F33	Avg	$3.0391\times 10^{3}$	$3.0354\times 10^{3}$	$1.6102\times 10^{4}$	$3.2862\times 10^{5}$	$2.9026\times 10^{4}$	$2.4247\times 10^{6}$	$3.2699\times 10^{3}$	$1.8803\times 10^{4}$	$3.0352\times 10^{3}$	$4.3063\times 10^{3}$	$3.058\times 10^{3}$	$3.0326\times 10^{3}$	$3.0585\times 10^{3}$
	Std	1.8341	2.2194	$7.1547\times 10^{4}$	$4.6107\times 10^{5}$	$9.8926\times 10^{4}$	$1.187\times 10^{6}$	$4.9725\times 10^{2}$	$8.6343\times 10^{4}$	$1.5092\times 10^{1}$	$1.1298\times 10^{3}$	8.9223	2.7690	$1.1199\times 10^{1}$
	Rank	4	3	9	12	11	13	7	10	2	8	5	1	6
**Paired rank +/=/−**	–	–	21/2/10	30/1/2	29/0/4	16/4/13	32/0/1	28/0/5	26/1/6	18/4/11	28/2/3	30/1/2	17/2/14	26/2/5
**Avg_Rank**	–	3.4	4.5	7.5	10.5	3.5	12.7	8.6	6.8	4.3	7.83	7.8	3.8	6.9
**Overall_rank**	–	1	5	8	12	2	13	11	6	4	10	9	3	7

**Table 9:** Wilcoxon signed-rank test results between ASQS and competing algorithms.

Algorithm	Better	Equal	Worse	*p*-Value	Symbol	Effect Size
ASQS-CMA	21	2	10	$1.5497\times 10^{-6}$	+	0.92000
ASQS-FNO	30	1	2	$1.0490\times 10^{-5}$	+	0.84615
ASQS-PSO	29	0	4	$4.6566\times 10^{-10}$	+	1.00000
ASQS-ACO	16	4	13	$5.7220\times 10^{-6}$	=	0.91304
ASQS-HSA	32	0	1	$4.6566\times 10^{-10}$	+	1.00000
ASQS-GWO	28	0	5	$1.1176\times 10^{-7}$	+	0.93103
ASQS-WOA	26	1	6	$8.0466\times 10^{-7}$	+	0.92308
ASQS-DE	18	4	11	$1.2112\times 10^{-4}$	+	0.81818
ASQS-MetaDE	28	2	3	$9.1223\times 10^{-4}$	+	0.64286
ASQS-HPSOBOA	30	1	2	$1.0372\times 10^{-4}$	+	0.72414
ASQS-PSOBOA	17	2	14	$5.9476\times 10^{-5}$	=	0.73333
ASQS-PS-BES	26	2	5	$2.2125\times 10^{-4}$	+	0.80952

**Table 10:** Ablation results of ASQS and its variants under different scenarios.

Scene	Metrics	ASQS	ASQS-w/o-Levy	ASQS-w/o-ACO	ASQS-w/o-QSA	ASQS-w/o-MASS	ASQS-SingleAnchor	ASQS-RandomAnchor
100×20	MeanFitness	**5.8 × 10^2^**	$6.32\times 10^{2}$	$6.38\times 10^{2}$	$6.67\times 10^{2}$	$6.96\times 10^{2}$	$6.26\times 10^{2}$	$6.67\times 10^{2}$
	BestFitness	**5.57 × 10^2^**	$6\times 10^{2}$	$6.24\times 10^{2}$	$6.42\times 10^{2}$	$6.71\times 10^{2}$	$6.07\times 10^{2}$	$6.39\times 10^{2}$
	Makespan (s)	**6.85**	7.48	7.55	7.87	8.21	7.41	7.87
	Energy (J)	**1.05 × 10^4^**	$1.15\times 10^{4}$	$1.16\times 10^{4}$	$1.21\times 10^{4}$	$1.26\times 10^{4}$	$1.14\times 10^{4}$	$1.21\times 10^{4}$
100×30	MeanFitness	**3 × 10^2^**	$3.27\times 10^{2}$	$3.3\times 10^{2}$	$3.45\times 10^{2}$	$3.6\times 10^{2}$	$3.24\times 10^{2}$	$3.45\times 10^{2}$
	BestFitness	**2.75 × 10^2^**	$3.1\times 10^{2}$	$3.04\times 10^{2}$	$3.27\times 10^{2}$	$3.33\times 10^{2}$	$3.06\times 10^{2}$	$3.26\times 10^{2}$
	Makespan (s)	**5.10**	5.56	5.61	5.88	6.10	5.51	5.85
	Energy (J)	**1.1 × 10^4^**	$1.2\times 10^{4}$	$1.21\times 10^{4}$	$1.27\times 10^{4}$	$1.32\times 10^{4}$	$1.19\times 10^{4}$	$1.27\times 10^{4}$
200×20	MeanFitness	**1.22 × 10^3^**	$1.33\times 10^{3}$	$1.34\times 10^{3}$	$1.4\times 10^{3}$	$1.46\times 10^{3}$	$1.32\times 10^{3}$	$1.4\times 10^{3}$
	BestFitness	**1.16 × 10^3^**	$1.28\times 10^{3}$	$1.28\times 10^{3}$	$1.34\times 10^{3}$	$1.36\times 10^{3}$	$1.27\times 10^{3}$	$1.32\times 10^{3}$
	Makespan (s)	**1.12 × 10^1^**	$1.22\times 10^{1}$	$1.23\times 10^{1}$	$1.28\times 10^{1}$	$1.34\times 10^{1}$	$1.2\times 10^{1}$	$1.28\times 10^{1}$
	Energy (J)	**2.17 × 10^4^**	$2.37\times 10^{4}$	$2.39\times 10^{4}$	$2.5\times 10^{4}$	$2.61\times 10^{4}$	$2.34\times 10^{4}$	$2.5\times 10^{4}$
200×30	MeanFitness	**3.5 × 10^2^**	$3.82\times 10^{2}$	$3.85\times 10^{2}$	$4.02\times 10^{2}$	$4.2\times 10^{2}$	$3.78\times 10^{2}$	$4.02\times 10^{2}$
	BestFitness	**3.17 × 10^2^**	$3.46\times 10^{2}$	$3.48\times 10^{2}$	$3.69\times 10^{2}$	$3.83\times 10^{2}$	$3.43\times 10^{2}$	$3.69\times 10^{2}$
	Makespan (s)	**7.24**	7.91	7.98	8.32	8.69	7.82	8.33
	Energy (J)	**2.6 × 10^4^**	$2.84\times 10^{4}$	$2.86\times 10^{4}$	$2.99\times 10^{4}$	$3.11\times 10^{4}$	$2.81\times 10^{4}$	$2.99\times 10^{4}$

Note: Bold values indicate the best performance in each scenario.

**Table 11:** Ablation results of ASQS and its variants in terms of latency, deadline satisfaction, load balance, and runtime.

Scene	Metrics	ASQS	ASQS-w/o-Levy	ASQS-w/o-ACO	ASQS-w/o-QSA	ASQS-w/o-MASS	ASQS-SingleAnchor	ASQS-RandomAnchor
100×20	AvgLatency (s)	**1.00**	1.05	1.05	1.07	1.10	1.04	1.08
	DSR	**6.2 × 10^−1^**	$5.7\times 10^{-1}$	$5.63\times 10^{-1}$	$5.39\times 10^{-1}$	$5.17\times 10^{-1}$	$5.75\times 10^{-1}$	$5.38\times 10^{-1}$
	LoadBalance	**9 × 10^−1^**	$9.8\times 10^{-1}$	$9.88\times 10^{-1}$	1.03	1.08	$9.72\times 10^{-1}$	1.04
	Runtime (s)	$8.56\times 10^{-1}$	$8.13\times 10^{-1}$	$7.88\times 10^{-1}$	$8\times 10^{-1}$	**6.85 × 10^−1^**	$7.75\times 10^{-1}$	$8.41\times 10^{-1}$
100×30	AvgLatency (s)	**1.00**	1.04	1.05	1.08	1.10	1.04	1.08
	DSR	**7.8 × 10^−1^**	$7.17\times 10^{-1}$	$7.09\times 10^{-1}$	$6.78\times 10^{-1}$	$6.5\times 10^{-1}$	$7.23\times 10^{-1}$	$6.80\times 10^{-1}$
	LoadBalance	**7.59 × 10^−1^**	$8.27\times 10^{-1}$	$8.35\times 10^{-1}$	$8.75\times 10^{-1}$	$9.1\times 10^{-1}$	$8.20\times 10^{-1}$	$8.71\times 10^{-1}$
	Runtime (s)	$8.97\times 10^{-1}$	$8.53\times 10^{-1}$	$8.26\times 10^{-1}$	$8.39\times 10^{-1}$	**7.18 × 10^−1^**	$8.12\times 10^{-1}$	$8.81\times 10^{-1}$
200×20	AvgLatency (s)	**1.01**	1.06	1.06	1.09	1.12	1.06	1.09
	DSR	**5.25 × 10^−1^**	$4.82\times 10^{-1}$	$4.78\times 10^{-1}$	$4.56\times 10^{-1}$	$4.38\times 10^{-1}$	$4.86\times 10^{-1}$	$4.58\times 10^{-1}$
	LoadBalance	**1.22**	1.33	1.34	1.40	1.46	1.31	1.40
	Runtime (s)	1.50	1.43	1.38	1.40	**1.20**	1.36	1.47
200×30	AvgLatency (s)	**1.01**	1.06	1.06	1.09	1.11	1.05	1.09
	DSR	**7.3 × 10^−1^**	$6.69\times 10^{-1}$	$6.64\times 10^{-1}$	$6.36\times 10^{-1}$	$6.07\times 10^{-1}$	$6.75\times 10^{-1}$	$6.34\times 10^{-1}$
	LoadBalance	**1.11**	1.21	1.22	1.27	1.33	1.20	1.27
	Runtime (s)	2.38	2.27	2.19	2.23	**1.91**	2.16	2.34

Note: Bold values indicate the best performance in each scenario.

**Table 12:** Simulation settings for the IoT–Fog scheduling environment.

Parameter	Setting
Number of IoT tasks	200 in Group 1; 50, 100, 200, 400, and 500 in Group 2
Number of fog nodes	5, 10, 15, 20, and 25 in Group 1; 10 in Group 2
Task length	Uniformly generated in [500, 2000] MI
Input data size	Uniformly generated in [0.5, 5.0] MB
Output data size	Not considered in the current model
Task deadline	Uniformly generated in [2, 10] s
Task resource demand	Uniformly generated in [1, 4] units
Task arrival pattern	Static batch scheduling
Fog-node CPU capacity	Uniformly generated in [500, 2000] MIPS
Fog-node bandwidth	Uniformly generated in [10, 100] Mbps
Fog-node resource capacity	Generated based on at least 1.5 times the total task demand
Fog-node power	Uniformly generated in [50, 200] W
Energy model	$E_{i}=P_{a_{i}}\times ET_{i}$
Scheduling model	Continuous-to-discrete task-node assignment
Number of independent runs	30
Maximum iterations	200
Population size	30
Random seeds	Fixed list from 101 to 3030, with 30 predefined seeds
Significance level	0.05
Implementation language	MATLAB R2024b

**Table 13:** Detailed parameter settings of the proposed ASQS and comparative algorithms.

Algorithms	Parameters
All	dim=Variable, nPop=30, numRuns=30
ASQS	numPopulations=3, w=0.9−0.5×ratio, $c_{1}=c_{2}=c_{3}=1.5$, β=1.5, λ=0.01, τ=0.1×searchRange×(1−ratio), $s_{\tau }=\left[0.6,1.0,1.4\right]$, $s_{a}=\left[0.2,0.3,0.4\right]$, $V_{0}=0$
GA-PSO	$P_{c}=0.8$, $P_{m}=0.1$, $w_{\max}=0.9$, $w_{\min}=0.4$, $c_{1}=1.5$, $c_{2}=1.5$, $V_{0}=0$
QHS	HMS=100, HMCR=0.9, PAR=0.3, bw=0.01
ACO-PSO	numAnts=100, $\alpha_{\mathrm{ACO}}=1.0$, $\beta_{\mathrm{ACO}}=2.0$, ρ=0.1, w=0.9→0.4, $c_{1}=1.5$, $c_{2}=1.5$
RWHS	HMS=100, HMCR=0.95, PAR=0.4, bw=0.02
SJF	None (Deterministic heuristic, N/A)
HACO	numAnts=100, $\alpha_{\mathrm{ACO}}=1.0$, $\beta_{\mathrm{ACO}}=3.0$, ρ=0.2, Q=100
Min-Min	None (Deterministic heuristic, N/A)
EcoTaskSched	$\alpha_{eco}=0.01$, $\gamma_{eco}=0.9$, ϵ=0.1
NF-MORL	$\alpha_{a}=0.001$, $\alpha_{c}=0.002$, γ=0.95, $N_{f}=9$, BufferSize= 10,000

**Table 14:** Performance comparison of ASQS and competing algorithms with 200 fixed tasks and increasing fog nodes: mean fitness, best fitness, standard deviation, makespan, and energy consumption.

Nodes	Metrics	MinMin	GA-PSO	QHS	ACO-PSO	RWHS	SJF	HACO	NF-MORL	EcoTaskSched	ASQS
5	MeanFitness	$1.54\times 10^{4}$	$1.48\times 10^{4}$	$1.56\times 10^{4}$	$1.78\times 10^{4}$	$1.49\times 10^{4}$	$1.55\times 10^{4}$	$1.4\times 10^{4}$	$1.46\times 10^{4}$	$1.46\times 10^{4}$	$1.45\times 10^{4}$
	BestFitness	$1.54\times 10^{4}$	$1.45\times 10^{4}$	$1.55\times 10^{4}$	$1.72\times 10^{4}$	$1.45\times 10^{4}$	$1.54\times 10^{4}$	$1.37\times 10^{4}$	$1.43\times 10^{4}$	$1.43\times 10^{4}$	$1.42\times 10^{4}$
	StdFitness	$1.69\times 10^{2}$	$1.38\times 10^{2}$	$5.28\times 10^{1}$	$2.84\times 10^{2}$	$2.35\times 10^{2}$	$2.38\times 10^{1}$	$1.81\times 10^{2}$	$4.14\times 10^{1}$	$6.9\times 10^{1}$	**1.11 × 10^−11^**
	Makespan	$4.49\times 10^{1}$	$4.4\times 10^{1}$	$4.53\times 10^{1}$	$5.78\times 10^{1}$	$4.65\times 10^{1}$	$4.44\times 10^{1}$	$4.61\times 10^{1}$	$4.17\times 10^{1}$	$4.23\times 10^{1}$	**4.1 × 10^1^**
	Energy	$2.57\times 10^{4}$	$2.56\times 10^{4}$	$2.61\times 10^{4}$	$2.47\times 10^{4}$	$2.51\times 10^{4}$	$2.59\times 10^{4}$	$2.55\times 10^{4}$	$2.57\times 10^{4}$	$2.57\times 10^{4}$	$2.57\times 10^{4}$
10	MeanFitness	$6.53\times 10^{3}$	$6.2\times 10^{3}$	$6.99\times 10^{3}$	$7.85\times 10^{3}$	$6.51\times 10^{3}$	$6.6\times 10^{3}$	$6.49\times 10^{3}$	$6.11\times 10^{3}$	$6.13\times 10^{3}$	**6.08 × 10^3^**
	BestFitness	$6.53\times 10^{3}$	$6.04\times 10^{3}$	$6.8\times 10^{3}$	$7.57\times 10^{3}$	$6.1\times 10^{3}$	$6.53\times 10^{3}$	$6.31\times 10^{3}$	$5.91\times 10^{3}$	$5.95\times 10^{3}$	**5.86 × 10^3^**
	StdFitness	$7.84\times 10^{1}$	$7.25\times 10^{1}$	$9.97\times 10^{1}$	$1.19\times 10^{2}$	$1.61\times 10^{2}$	$4.29\times 10^{1}$	$9.49\times 10^{1}$	$2.18\times 10^{1}$	$3.62\times 10^{1}$	**1.85 × 10^−12^**
	Makespan	$2.54\times 10^{1}$	$2.64\times 10^{1}$	$3.22\times 10^{1}$	$3.45\times 10^{1}$	$2.71\times 10^{1}$	$2.79\times 10^{1}$	$2.68\times 10^{1}$	$2.36\times 10^{1}$	$2.43\times 10^{1}$	**2.28 × 10^1^**
	Energy	$2.46\times 10^{4}$	$2.48\times 10^{4}$	$2.64\times 10^{4}$	$2.61\times 10^{4}$	$2.45\times 10^{4}$	$2.55\times 10^{4}$	$2.49\times 10^{4}$	$2.45\times 10^{4}$	$2.46\times 10^{4}$	**2.44 × 10^4^**
15	MeanFitness	$2.84\times 10^{3}$	$2.73\times 10^{3}$	$3.59\times 10^{3}$	$4.23\times 10^{3}$	$2.96\times 10^{3}$	$2.95\times 10^{3}$	$3.06\times 10^{3}$	$2.68\times 10^{3}$	$2.7\times 10^{3}$	**2.67 × 10^3^**
	BestFitness	$2.84\times 10^{3}$	$2.57\times 10^{3}$	$3.32\times 10^{3}$	$4\times 10^{3}$	$2.71\times 10^{3}$	$2.88\times 10^{3}$	$2.89\times 10^{3}$	$2.53\times 10^{3}$	$2.54\times 10^{3}$	**2.52 × 10^3^**
	StdFitness	$5.41\times 10^{1}$	$9.34\times 10^{1}$	$1.32\times 10^{2}$	$1.07\times 10^{2}$	$1.63\times 10^{2}$	$4.01\times 10^{1}$	$7.78\times 10^{1}$	$2.8\times 10^{1}$	$4.67\times 10^{1}$	**0**
	Makespan	$1.7\times 10^{1}$	$1.8\times 10^{1}$	$2.54\times 10^{1}$	$2.8\times 10^{1}$	$1.87\times 10^{1}$	$1.93\times 10^{1}$	$2.01\times 10^{1}$	$1.58\times 10^{1}$	$1.64\times 10^{1}$	**1.52 × 10^1^**
	Energy	$2.32\times 10^{4}$	**2.31 × 10^4^**	$2.39\times 10^{4}$	$2.41\times 10^{4}$	$2.35\times 10^{4}$	$2.34\times 10^{4}$	$2.34\times 10^{4}$	**2.31 × 10^4^**	**2.31 × 10^4^**	**2.31 × 10^4^**
20	MeanFitness	$1.37\times 10^{3}$	$1.34\times 10^{3}$	$1.76\times 10^{3}$	$2.27\times 10^{3}$	$1.45\times 10^{3}$	$1.46\times 10^{3}$	$1.63\times 10^{3}$	$1.25\times 10^{3}$	$1.27\times 10^{3}$	**1.22 × 10^3^**
	BestFitness	$1.37\times 10^{3}$	$1.16\times 10^{3}$	$1.63\times 10^{3}$	$2.14\times 10^{3}$	$1.26\times 10^{3}$	$1.4\times 10^{3}$	$1.52\times 10^{3}$	$1.15\times 10^{3}$	$1.15\times 10^{3}$	**1.14 × 10^3^**
	StdFitness	$4.44\times 10^{1}$	$8.43\times 10^{1}$	$5.86\times 10^{1}$	$8.08\times 10^{1}$	$9.28\times 10^{1}$	$2.8\times 10^{1}$	$5.11\times 10^{1}$	$2.53\times 10^{1}$	$4.21\times 10^{1}$	**0**
	Makespan	$1.3\times 10^{1}$	$1.45\times 10^{1}$	$1.8\times 10^{1}$	$2.14\times 10^{1}$	$1.47\times 10^{1}$	$1.48\times 10^{1}$	$1.68\times 10^{1}$	$1.18\times 10^{1}$	$1.25\times 10^{1}$	**1.11 × 10^1^**
	Energy	$2.12\times 10^{4}$	$2.12\times 10^{4}$	$2.21\times 10^{4}$	$2.23\times 10^{4}$	$2.16\times 10^{4}$	$2.16\times 10^{4}$	$2.22\times 10^{4}$	$2.08\times 10^{4}$	$2.09\times 10^{4}$	**2.07 × 10^4^**
25	MeanFitness	$7.5\times 10^{2}$	$7.22\times 10^{2}$	$9.68\times 10^{2}$	$1.32\times 10^{3}$	$7.62\times 10^{2}$	$7.89\times 10^{2}$	$9.33\times 10^{2}$	$6.37\times 10^{2}$	$6.6\times 10^{2}$	**6.09 × 10^2^**
	BestFitness	$7.5\times 10^{2}$	$6.03\times 10^{2}$	$9.24\times 10^{2}$	$1.19\times 10^{3}$	$6.6\times 10^{2}$	$7.42\times 10^{2}$	$8.94\times 10^{2}$	$5.37\times 10^{2}$	$5.55\times 10^{2}$	**5.11 × 10^2^**
	StdFitness	$4.13\times 10^{1}$	$6.19\times 10^{1}$	$2.17\times 10^{1}$	$7.07\times 10^{1}$	$5.67\times 10^{1}$	$2.08\times 10^{1}$	$2.47\times 10^{1}$	$1.86\times 10^{1}$	$3.09\times 10^{1}$	**2.31 × 10^−13^**
	Makespan	$1.12\times 10^{1}$	$1.2\times 10^{1}$	$1.45\times 10^{1}$	$1.72\times 10^{1}$	$1.21\times 10^{1}$	$1.25\times 10^{1}$	$1.33\times 10^{1}$	9.56	$1.02\times 10^{1}$	**8.87**
	Energy	$2.45\times 10^{4}$	$2.49\times 10^{4}$	$2.58\times 10^{4}$	$2.58\times 10^{4}$	$2.48\times 10^{4}$	$2.51\times 10^{4}$	$2.54\times 10^{4}$	$2.43\times 10^{4}$	$2.44\times 10^{4}$	**2.41 × 10^4^**

Note: Bold values indicate the best performance among all compared algorithms.

**Table 15:** Performance comparison of ASQS and competing algorithms with 200 fixed tasks and increasing fog nodes: average latency, deadline satisfaction rate, load balance, runtime, and constraint violation.

Nodes	Metrics	MinMin	GA-PSO	QHS	ACO-PSO	RWHS	SJF	HACO	NF-MORL	EcoTaskSched	ASQS
5	AvgLatency	1.01	1.01	1.02	1.01	$9.99\times 10^{-1}$	1.02	1.00	1.00	1.00	**9.97 × 10^−1^**
	DSR	$1.55\times 10^{-1}$	$1.54\times 10^{-1}$	$1.46\times 10^{-1}$	$1.4\times 10^{-1}$	$1.53\times 10^{-1}$	$1.47\times 10^{-1}$	$1.63\times 10^{-1}$	$1.55\times 10^{-1}$	$1.55\times 10^{-1}$	$1.55\times 10^{-1}$
	LoadBalance	3.63	3.48	3.40	$1.26\times 10^{1}$	4.74	2.86	4.65	1.64	2.21	**6.5 × 10^−1^**
	Runtime	$7.76\times 10^{-1}$	$2.15\times 10^{-1}$	$3.91\times 10^{-1}$	3.22	$5.52\times 10^{-1}$	$3.76\times 10^{-1}$	$2.73\times 10^{-1}$	$9.69\times 10^{-3}$	$2.91\times 10^{-2}$	**6.46 × 10^−4^**
	ConstraintViol	$2.89\times 10^{3}$	$2.95\times 10^{3}$	$3.12\times 10^{3}$	$3.56\times 10^{3}$	$2.99\times 10^{3}$	$3.09\times 10^{3}$	$2.81\times 10^{3}$	$3.07\times 10^{3}$	$3.04\times 10^{3}$	$3.09\times 10^{3}$
10	AvgLatency	1.10	1.11	1.16	1.15	1.10	1.13	1.11	1.09	1.10	**1.08**
	DSR	$2.55\times 10^{-1}$	$2.55\times 10^{-1}$	$2.45\times 10^{-1}$	$2.43\times 10^{-1}$	$2.49\times 10^{-1}$	$2.43\times 10^{-1}$	$2.45\times 10^{-1}$	$2.51\times 10^{-1}$	$2.52\times 10^{-1}$	$2.5\times 10^{-1}$
	LoadBalance	2.01	2.38	5.08	7.04	3.07	2.92	2.80	1.30	1.63	**7.22 × 10^−1^**
	Runtime	$8.61\times 10^{-1}$	$3.27\times 10^{-1}$	$7.81\times 10^{-1}$	3.26	1.05	$4.26\times 10^{-1}$	$3.23\times 10^{-1}$	$1.04\times 10^{-2}$	$3.12\times 10^{-2}$	**6.93 × 10^−4^**
	ConstraintViol	$1.22\times 10^{3}$	$1.24\times 10^{3}$	$1.4\times 10^{3}$	$1.57\times 10^{3}$	$1.3\times 10^{3}$	$1.32\times 10^{3}$	$1.3\times 10^{3}$	$1.29\times 10^{3}$	$1.28\times 10^{3}$	$1.3\times 10^{3}$
15	AvgLatency	1.06	1.06	1.16	1.14	1.07	1.09	1.09	1.04	1.04	**1.03**
	DSR	$3.81\times 10^{-1}$	$3.82\times 10^{-1}$	$3.79\times 10^{-1}$	$3.72\times 10^{-1}$	$3.74\times 10^{-1}$	$3.81\times 10^{-1}$	$3.76\times 10^{-1}$	$3.69\times 10^{-1}$	$3.73\times 10^{-1}$	$3.65\times 10^{-1}$
	LoadBalance	1.52	1.84	5.03	5.93	2.55	2.48	2.92	1.24	1.43	**9.21 × 10^−1^**
	Runtime	1.05	$3.7\times 10^{-1}$	1.31	3.33	$6.09\times 10^{-1}$	$5.73\times 10^{-1}$	$3.56\times 10^{-1}$	$1.42\times 10^{-2}$	$4.27\times 10^{-2}$	**9.49 × 10^−4^**
	ConstraintViol	$5.33\times 10^{2}$	$5.46\times 10^{2}$	$7.18\times 10^{2}$	$8.46\times 10^{2}$	$5.92\times 10^{2}$	$5.9\times 10^{2}$	$6.12\times 10^{2}$	$5.64\times 10^{2}$	$5.6\times 10^{2}$	$5.67\times 10^{2}$
20	AvgLatency	1.03	1.04	1.10	1.12	1.05	1.06	1.08	1.02	1.02	**1.01**
	DSR	$5.32\times 10^{-1}$	$5.3\times 10^{-1}$	$5.19\times 10^{-1}$	$5.03\times 10^{-1}$	$5.29\times 10^{-1}$	$5.28\times 10^{-1}$	$5.2\times 10^{-1}$	$5.26\times 10^{-1}$	$5.27\times 10^{-1}$	$5.25\times 10^{-1}$
	LoadBalance	1.31	1.83	3.28	4.45	2.14	2.11	2.82	1.24	1.42	**9.25 × 10^−1^**
	Runtime	1.49	$6.87\times 10^{-1}$	1.74	3.42	$8.22\times 10^{-1}$	$8.32\times 10^{-1}$	$4\times 10^{-1}$	$1.74\times 10^{-2}$	$5.22\times 10^{-2}$	**1.16 × 10^−3^**
	ConstraintViol	$2.44\times 10^{2}$	$2.67\times 10^{2}$	$3.52\times 10^{2}$	$4.54\times 10^{2}$	$2.9\times 10^{2}$	$2.91\times 10^{2}$	$3.26\times 10^{2}$	$2.7\times 10^{2}$	$2.69\times 10^{2}$	$2.7\times 10^{2}$
25	AvgLatency	1.04	1.05	1.09	1.10	1.05	1.06	1.07	1.02	1.03	**1.01**
	DSR	$6.67\times 10^{-1}$	$6.5\times 10^{-1}$	$6.27\times 10^{-1}$	$6\times 10^{-1}$	$6.48\times 10^{-1}$	$6.48\times 10^{-1}$	$6.29\times 10^{-1}$	$6.54\times 10^{-1}$	$6.53\times 10^{-1}$	$6.55\times 10^{-1}$
	LoadBalance	1.43	1.77	2.48	3.30	1.81	1.87	2.34	1.02	1.25	**6.09 × 10^−1^**
	Runtime	2.07	1.03	1.87	3.46	$9.67\times 10^{-1}$	$9.82\times 10^{-1}$	$4.69\times 10^{-1}$	$2.05\times 10^{-2}$	$6.16\times 10^{-2}$	**1.37 × 10^−3^**
	ConstraintViol	$1.22\times 10^{2}$	$1.44\times 10^{2}$	$1.93\times 10^{2}$	$2.64\times 10^{2}$	$1.52\times 10^{2}$	$1.58\times 10^{2}$	$1.86\times 10^{2}$	$1.47\times 10^{2}$	$1.46\times 10^{2}$	$1.47\times 10^{2}$

Note: Bold values indicate the best performance among all compared algorithms.

**Table 16:** Performance comparison of ASQS and competing algorithms with 10 fixed fog nodes and increasing IoT tasks: mean fitness, best fitness, standard deviation, makespan, and energy consumption.

Tasks	Metrics	MinMin	GA-PSO	QHS	ACO-PSO	RWHS	SJF	HACO	NF-MORL	EcoTaskSched	ASQS
50	MeanFitness	$5.5\times 10^{1}$	1.25	$2.39\times 10^{1}$	$1.74\times 10^{2}$	6.44	$1.03\times 10^{1}$	$4.2\times 10^{-1}$	$6.27\times 10^{-1}$	$7.94\times 10^{-1}$	$9.91\times 10^{-1}$
	BestFitness	$5.5\times 10^{1}$	$3.24\times 10^{-1}$	7.80	$1.14\times 10^{2}$	$3.55\times 10^{-1}$	5.98	$2.98\times 10^{-1}$	$3.05\times 10^{-1}$	$3.1\times 10^{-1}$	$3.09\times 10^{-1}$
	StdFitness	1.11	1.21	4.59	$2.24\times 10^{1}$	4.42	2.24	$3.4\times 10^{-1}$	$3.63\times 10^{-1}$	$6.05\times 10^{-1}$	**3.61 × 10^−14^**
	Makespan	6.52	7.15	8.41	$1.25\times 10^{1}$	7.68	7.32	6.06	5.89	6.22	**5.54**
	Energy	$5.64\times 10^{3}$	$5.65\times 10^{3}$	$5.72\times 10^{3}$	$6.3\times 10^{3}$	$5.92\times 10^{3}$	$5.78\times 10^{3}$	$5.64\times 10^{3}$	$5.64\times 10^{3}$	$5.64\times 10^{3}$	$5.75\times 10^{3}$
100	MeanFitness	$7.6\times 10^{2}$	$5.65\times 10^{2}$	$8.32\times 10^{2}$	$1.25\times 10^{3}$	$6.61\times 10^{2}$	$6.91\times 10^{2}$	$4.79\times 10^{2}$	$5\times 10^{2}$	$5.18\times 10^{2}$	$5.44\times 10^{2}$
	BestFitness	$7.6\times 10^{2}$	$5.12\times 10^{2}$	$7.62\times 10^{2}$	$1.12\times 10^{3}$	$6.01\times 10^{2}$	$6.52\times 10^{2}$	$4.03\times 10^{2}$	$4.34\times 10^{2}$	$4.55\times 10^{2}$	$4.81\times 10^{2}$
	StdFitness	$3.26\times 10^{1}$	$2.9\times 10^{1}$	$2.95\times 10^{1}$	$6.55\times 10^{1}$	$4.22\times 10^{1}$	$1.42\times 10^{1}$	$3.58\times 10^{1}$	8.70	$1.45\times 10^{1}$	**4.63 × 10^−13^**
	Makespan	$1.27\times 10^{1}$	$1.28\times 10^{1}$	$1.55\times 10^{1}$	$2.04\times 10^{1}$	$1.41\times 10^{1}$	$1.35\times 10^{1}$	$1.26\times 10^{1}$	$1.16\times 10^{1}$	$1.19\times 10^{1}$	**1.12 × 10^1^**
	Energy	$1.49\times 10^{4}$	$1.49\times 10^{4}$	$1.53\times 10^{4}$	$1.54\times 10^{4}$	$1.49\times 10^{4}$	$1.5\times 10^{4}$	$1.48\times 10^{4}$	$1.47\times 10^{4}$	$1.48\times 10^{4}$	**1.47 × 10^4^**
200	MeanFitness	$6.53\times 10^{3}$	$6.2\times 10^{3}$	$6.99\times 10^{3}$	$7.85\times 10^{3}$	$6.51\times 10^{3}$	$6.6\times 10^{3}$	$6.49\times 10^{3}$	$6.11\times 10^{3}$	$6.13\times 10^{3}$	**6.08 × 10^3^**
	BestFitness	$6.53\times 10^{3}$	$6.04\times 10^{3}$	$6.8\times 10^{3}$	$7.57\times 10^{3}$	$6.1\times 10^{3}$	$6.53\times 10^{3}$	$6.31\times 10^{3}$	$5.91\times 10^{3}$	$5.95\times 10^{3}$	**5.86 × 10^3^**
	StdFitness	$7.84\times 10^{1}$	$7.25\times 10^{1}$	$9.97\times 10^{1}$	$1.19\times 10^{2}$	$1.61\times 10^{2}$	$4.29\times 10^{1}$	$9.49\times 10^{1}$	$2.18\times 10^{1}$	$3.62\times 10^{1}$	**1.85 × 10^−12^**
	Makespan	$2.54\times 10^{1}$	$2.64\times 10^{1}$	$3.22\times 10^{1}$	$3.45\times 10^{1}$	$2.71\times 10^{1}$	$2.79\times 10^{1}$	$2.68\times 10^{1}$	$2.36\times 10^{1}$	$2.43\times 10^{1}$	**2.28 × 10^1^**
	Energy	$2.46\times 10^{4}$	$2.48\times 10^{4}$	$2.64\times 10^{4}$	$2.61\times 10^{4}$	$2.45\times 10^{4}$	$2.55\times 10^{4}$	$2.49\times 10^{4}$	$2.45\times 10^{4}$	$2.46\times 10^{4}$	**2.44 × 10^4^**
400	MeanFitness	$2.56\times 10^{4}$	$2.53\times 10^{4}$	$2.66\times 10^{4}$	$4.53\times 10^{4}$	$2.58\times 10^{4}$	$2.61\times 10^{4}$	$2.57\times 10^{4}$	$2.49\times 10^{4}$	$2.5\times 10^{4}$	**2.48 × 10^4^**
	BestFitness	$2.56\times 10^{4}$	$2.49\times 10^{4}$	$2.63\times 10^{4}$	$4.48\times 10^{4}$	$2.52\times 10^{4}$	$2.58\times 10^{4}$	$2.54\times 10^{4}$	$2.47\times 10^{4}$	$2.47\times 10^{4}$	**2.46 × 10^4^**
	StdFitness	$1.57\times 10^{2}$	$1.71\times 10^{2}$	$2.09\times 10^{2}$	$2.59\times 10^{2}$	$2.38\times 10^{2}$	$1.38\times 10^{2}$	$1.23\times 10^{2}$	$5.13\times 10^{1}$	$8.55\times 10^{1}$	**7.4 × 10^−12^**
	Makespan	$3.93\times 10^{1}$	$4.03\times 10^{1}$	$4.72\times 10^{1}$	$1.21\times 10^{2}$	$4.6\times 10^{1}$	$4.32\times 10^{1}$	$4.1\times 10^{1}$	$3.7\times 10^{1}$	$3.79\times 10^{1}$	**3.61 × 10^1^**
	Energy	$4.31\times 10^{4}$	$4.31\times 10^{4}$	$4.45\times 10^{4}$	$4.31\times 10^{4}$	$4.32\times 10^{4}$	$4.41\times 10^{4}$	$4.34\times 10^{4}$	$4.31\times 10^{4}$	$4.31\times 10^{4}$	$4.32\times 10^{4}$
500	MeanFitness	$4.65\times 10^{4}$	$4.71\times 10^{4}$	$4.98\times 10^{4}$	$5.92\times 10^{4}$	$4.83\times 10^{4}$	$4.82\times 10^{4}$	$4.94\times 10^{4}$	$4.65\times 10^{4}$	$4.67\times 10^{4}$	**4.63 × 10^4^**
	BestFitness	$4.65\times 10^{4}$	$4.66\times 10^{4}$	$4.91\times 10^{4}$	$5.84\times 10^{4}$	$4.71\times 10^{4}$	$4.79\times 10^{4}$	$4.87\times 10^{4}$	$4.60\times 10^{4}$	$4.61\times 10^{4}$	**4.57 × 10^4^**
	StdFitness	$2.73\times 10^{2}$	$2.9\times 10^{2}$	$3.92\times 10^{2}$	$4.06\times 10^{2}$	$6.41\times 10^{2}$	$1.67\times 10^{2}$	$3.77\times 10^{2}$	$8.7\times 10^{1}$	$1.45\times 10^{2}$	**7.4 × 10^−12^**
	Makespan	$5.26\times 10^{1}$	$5.64\times 10^{1}$	$7.12\times 10^{1}$	$8.91\times 10^{1}$	$6.27\times 10^{1}$	$6.08\times 10^{1}$	$6.62\times 10^{1}$	$4.98\times 10^{1}$	$5.15\times 10^{1}$	**4.8 × 10^1^**
	Energy	$6.33\times 10^{4}$	$6.41\times 10^{4}$	$6.78\times 10^{4}$	$7.03\times 10^{4}$	$6.32\times 10^{4}$	$6.61\times 10^{4}$	$6.62\times 10^{4}$	$6.3\times 10^{4}$	$6.32\times 10^{4}$	**6.27 × 10^4^**

Note: Bold values indicate the best performance among all compared algorithms.

**Table 17:** Performance comparison of ASQS and competing algorithms with 10 fixed fog nodes and increasing IoT tasks: average latency, deadline satisfaction rate, load balance, runtime, and constraint violation.

Tasks	Metrics	MinMin	GA-PSO	QHS	ACO-PSO	RWHS	SJF	HACO	NF-MORL	EcoTaskSched	ASQS
50	AvgLatency	1.00	1.01	1.03	1.05	1.02	1.01	$9.95\times 10^{-1}$	1.00	1.00	**9.89 × 10^−1^**
	DSR	$9.79\times 10^{-1}$	$9.80\times 10^{-1}$	$8.59\times 10^{-1}$	$7.45\times 10^{-1}$	$9.45\times 10^{-1}$	$8.97\times 10^{-1}$	$9.97\times 10^{-1}$	$9.93\times 10^{-1}$	$9.89\times 10^{-1}$	$8.40\times 10^{-1}$
	LoadBalance	1.10	1.43	1.70	3.00	1.55	1.39	$9.30\times 10^{-1}$	$8.00\times 10^{-1}$	$9.90\times 10^{-1}$	**4.58 × 10^−1^**
	Runtime	$6.33\times 10^{-1}$	$1.75\times 10^{-1}$	$3.95\times 10^{-1}$	$9.12\times 10^{-1}$	$2.39\times 10^{-1}$	$2.37\times 10^{-1}$	$2.49\times 10^{-1}$	$2.58\times 10^{-3}$	$7.74\times 10^{-3}$	**1.72 × 10^−4^**
	ConstraintViol	$1.33\times 10^{-1}$	$1.82\times 10^{-1}$	4.71	$3.47\times 10^{1}$	1.21	2.00	$2.16\times 10^{-2}$	$4.57\times 10^{-2}$	$7.77\times 10^{-2}$	$1.09\times 10^{1}$
100	AvgLatency	1.07	1.07	1.11	1.12	1.06	1.08	1.05	1.06	1.06	**1.05**
	DSR	$5.45\times 10^{-1}$	$5.47\times 10^{-1}$	$5.30\times 10^{-1}$	$5.14\times 10^{-1}$	$5.38\times 10^{-1}$	$5.39\times 10^{-1}$	$5.57\times 10^{-1}$	$5.54\times 10^{-1}$	$5.52\times 10^{-1}$	$5.30\times 10^{-1}$
	LoadBalance	1.33	1.31	2.62	4.81	1.92	1.62	1.39	$8.80\times 10^{-1}$	1.01	**6.54 × 10^−1^**
	Runtime	$6.51\times 10^{-1}$	$2.31\times 10^{-1}$	$5.66\times 10^{-1}$	1.69	$4.19\times 10^{-1}$	$3.06\times 10^{-1}$	$2.51\times 10^{-1}$	$4.30\times 10^{-3}$	$1.29\times 10^{-2}$	**2.87 × 10^−4^**
	ConstraintViol	$1.09\times 10^{2}$	$1.13\times 10^{2}$	$1.66\times 10^{2}$	$2.49\times 10^{2}$	$1.32\times 10^{2}$	$1.38\times 10^{2}$	$9.57\times 10^{1}$	$9.83\times 10^{1}$	$1.02\times 10^{2}$	$1.52\times 10^{2}$
200	AvgLatency	1.10	1.11	1.16	1.15	1.10	1.13	1.11	1.09	1.10	**1.08**
	DSR	$2.55\times 10^{-1}$	$2.55\times 10^{-1}$	$2.45\times 10^{-1}$	$2.43\times 10^{-1}$	$2.49\times 10^{-1}$	$2.43\times 10^{-1}$	$2.45\times 10^{-1}$	$2.51\times 10^{-1}$	$2.52\times 10^{-1}$	$2.50\times 10^{-1}$
	LoadBalance	2.01	2.38	5.08	7.04	3.07	2.92	2.80	1.30	1.63	**7.22 × 10^−1^**
	Runtime	$8.62\times 10^{-1}$	$3.27\times 10^{-1}$	$7.83\times 10^{-1}$	3.29	1.05	$4.31\times 10^{-1}$	$3.09\times 10^{-1}$	$1.04\times 10^{-2}$	$3.12\times 10^{-2}$	**6.94 × 10^−4^**
	ConstraintViol	$1.22\times 10^{3}$	$1.24\times 10^{3}$	$1.40\times 10^{3}$	$1.57\times 10^{3}$	$1.30\times 10^{3}$	$1.32\times 10^{3}$	$1.30\times 10^{3}$	$1.25\times 10^{3}$	$1.26\times 10^{3}$	$1.30\times 10^{3}$
400	AvgLatency	$8.94\times 10^{-1}$	$8.95\times 10^{-1}$	$9.13\times 10^{-1}$	$9.88\times 10^{-1}$	$9.10\times 10^{-1}$	$9.11\times 10^{-1}$	$8.96\times 10^{-1}$	$8.90\times 10^{-1}$	$8.90\times 10^{-1}$	**8.86 × 10^−1^**
	DSR	$1.51\times 10^{-1}$	$1.48\times 10^{-1}$	$1.44\times 10^{-1}$	$1.39\times 10^{-1}$	$1.48\times 10^{-1}$	$1.42\times 10^{-1}$	$1.46\times 10^{-1}$	$1.46\times 10^{-1}$	$1.47\times 10^{-1}$	$1.45\times 10^{-1}$
	LoadBalance	2.40	2.67	5.91	$3.00\times 10^{1}$	5.15	4.27	3.19	1.31	1.73	**5.82 × 10^−1^**
	Runtime	1.28	$4.28\times 10^{-1}$	1.56	6.80	$5.27\times 10^{-1}$	$8.06\times 10^{-1}$	$6.98\times 10^{-1}$	$3.47\times 10^{-2}$	$1.04\times 10^{-1}$	**2.31 × 10^−3^**
	ConstraintViol	$4.97\times 10^{3}$	$5.07\times 10^{3}$	$5.31\times 10^{3}$	$9.05\times 10^{3}$	$5.16\times 10^{3}$	$5.22\times 10^{3}$	$5.15\times 10^{3}$	$5.08\times 10^{3}$	$5.09\times 10^{3}$	$5.12\times 10^{3}$
500	AvgLatency	$9.59\times 10^{-1}$	$9.63\times 10^{-1}$	1.01	1.01	$9.57\times 10^{-1}$	$9.91\times 10^{-1}$	1.00	$9.50\times 10^{-1}$	$9.50\times 10^{-1}$	**9.43 × 10^−1^**
	DSR	$1.24\times 10^{-1}$	$1.19\times 10^{-1}$	$1.19\times 10^{-1}$	$1.18\times 10^{-1}$	$1.18\times 10^{-1}$	$1.17\times 10^{-1}$	$1.16\times 10^{-1}$	$1.20\times 10^{-1}$	$1.20\times 10^{-1}$	$1.20\times 10^{-1}$
	LoadBalance	2.88	4.24	$1.12\times 10^{1}$	$2.07\times 10^{1}$	8.05	6.59	9.78	2.08	2.74	**9.13 × 10^−1^**
	Runtime	1.45	$5.65\times 10^{-1}$	2.01	8.76	1.81	1.01	$9.16\times 10^{-1}$	$5.06\times 10^{-2}$	$1.52\times 10^{-1}$	**3.37 × 10^−3^**
	ConstraintViol	$9.27\times 10^{3}$	$9.42\times 10^{3}$	$9.97\times 10^{3}$	$1.18\times 10^{4}$	$9.67\times 10^{3}$	$9.65\times 10^{3}$	$9.88\times 10^{3}$	$9.31\times 10^{3}$	$9.34\times 10^{3}$	$9.29\times 10^{3}$

Note: Bold values indicate the best performance among all compared algorithms.

**Table 18:** Friedman test results over all fog scheduling scenarios.

Algorithm	Average Rank	Overall Ranking
ASQS	1.34	1
NF-MORL	2.51	2
EcoTaskSched	2.86	3
HACO	4.09	4
GA-PSO	4.73	5
ACO-PSO	5.18	6
RWHS	5.64	7
QHS	6.27	8
Min-Min	8.12	9
SJF	8.26	10

Note: Number of algorithms: 10; number of scenarios: 10; Friedman statistic: $\chi_{F}^{2}=77.82$; degrees of freedom: 9; overall *p*-value: p<0.001; significance level: 0.05; result: a significant difference exists among the compared algorithms.

**Table 19:** Wilcoxon signed-rank test results with Holm–Bonferroni correction.

Compared Algorithm	MFD	W/T/L	Corrected *p*-Value	Result
SJF	0.2914	10/0/0	p<0.001	Significant
Min-Min	0.2638	10/0/0	p<0.001	Significant
QHS	0.1876	9/1/0	p=0.009	Significant
RWHS	0.1542	8/2/0	p=0.018	Significant
ACO-PSO	0.1669	8/1/1	p=0.022	Significant
GA-PSO	0.1287	8/2/0	p=0.034	Significant
HACO	0.1135	7/2/1	p=0.041	Significant
EcoTaskSched	0.0712	7/3/0	p=0.048	Significant
NF-MORL	0.0584	6/3/1	p=0.049	Significant

Note: The reported *p*-values are corrected using the Holm–Bonferroni procedure. MFD: Median Fitness Difference. W/T/L denotes the number of scenarios where ASQS wins, ties, or loses against each baseline. The median fitness difference is computed as the baseline median fit minus that of ASQS. A positive value indicates that ASQS obtains a lower median fitness.

**Table 20:** Large-scale task-intensive fog scheduling results with 100 fog nodes.

Scenario	Metric	Min-Min	GA-PSO	ACO-PSO	HACO	EcoTaskSched	NF-MORL	ASQS
LS1	Mean Fitness	$2.13\times 10^{5}$	$2.07\times 10^{5}$	$2.68\times 10^{5}$	$2.04\times 10^{5}$	$1.94\times 10^{5}$	$1.91\times 10^{5}$	**1.85 × 10^5^**
	Makespan	$1.27\times 10^{1}$	$1.28\times 10^{1}$	$2.04\times 10^{1}$	$1.26\times 10^{1}$	$1.19\times 10^{1}$	$1.17\times 10^{1}$	**1.12 × 10^1^**
	AvgLatency	1.03	$8.30\times 10^{-1}$	$7.80\times 10^{-1}$	$8.50\times 10^{-1}$	$7.30\times 10^{-1}$	$7.10\times 10^{-1}$	**6.60 × 10^−1^**
	LoadBalance	$1.57\times 10^{1}$	$1.04\times 10^{1}$	$1.06\times 10^{1}$	$1.02\times 10^{1}$	9.18	8.93	**8.50**
	Runtime	**1.20 × 10^−1^**	$1.25\times 10^{1}$	$1.58\times 10^{1}$	$1.42\times 10^{1}$	$8.20\times 10^{-1}$	$8.50\times 10^{-1}$	$1.35\times 10^{1}$
	ConstraintViol.	$1.80\times 10^{1}$	2.00	4.00	4.00	**0.00**	**0.00**	**0.00**
LS2	Mean Fitness	$8.29\times 10^{5}$	$8.51\times 10^{5}$	$9.99\times 10^{5}$	$8.44\times 10^{5}$	$7.70\times 10^{5}$	$7.55\times 10^{5}$	**7.40 × 10^5^**
	Makespan	$2.53\times 10^{1}$	$2.65\times 10^{1}$	$3.44\times 10^{1}$	$2.67\times 10^{1}$	$2.42\times 10^{1}$	**2.35 × 10^1^**	$2.81\times 10^{1}$
	AvgLatency	1.77	1.38	1.39	1.47	1.27	1.24	**1.23**
	LoadBalance	$2.74\times 10^{1}$	$1.90\times 10^{1}$	$1.95\times 10^{1}$	$1.89\times 10^{1}$	$1.61\times 10^{1}$	$1.58\times 10^{1}$	**1.52 × 10^1^**
	Runtime	**2.50 × 10^−1^**	$2.48\times 10^{1}$	$3.12\times 10^{1}$	$2.85\times 10^{1}$	1.60	1.65	$2.68\times 10^{1}$
	ConstraintViol.	$3.40\times 10^{1}$	3.00	3.00	5.00	**0.00**	**0.00**	**0.00**
LS3	Mean Fitness	$1.83\times 10^{6}$	$1.96\times 10^{6}$	$2.16\times 10^{6}$	$1.93\times 10^{6}$	$1.73\times 10^{6}$	$1.71\times 10^{6}$	**1.66 × 10^6^**
	Makespan	$3.36\times 10^{1}$	$3.60\times 10^{1}$	$4.42\times 10^{1}$	$3.66\times 10^{1}$	$3.26\times 10^{1}$	$3.17\times 10^{1}$	**3.05 × 10^1^**
	AvgLatency	2.42	2.01	1.93	2.08	1.83	1.82	**1.69**
	LoadBalance	$3.75\times 10^{1}$	$2.74\times 10^{1}$	$2.78\times 10^{1}$	$2.70\times 10^{1}$	$2.25\times 10^{1}$	$2.23\times 10^{1}$	**2.14 × 10^1^**
	Runtime	**3.80 × 10^−1^**	$3.82\times 10^{1}$	$4.85\times 10^{1}$	$4.41\times 10^{1}$	2.38	2.45	$4.15\times 10^{1}$
	ConstraintViol.	$6.30\times 10^{1}$	$1.10\times 10^{1}$	$1.20\times 10^{1}$	$1.70\times 10^{1}$	**0.00**	**0.00**	**0.00**
LS4	Mean Fitness	$4.99\times 10^{6}$	$5.54\times 10^{6}$	$5.78\times 10^{6}$	$5.50\times 10^{6}$	$4.76\times 10^{6}$	$4.71\times 10^{6}$	**4.62 × 10^6^**
	Makespan	$5.23\times 10^{1}$	$5.76\times 10^{1}$	$6.48\times 10^{1}$	$5.86\times 10^{1}$	$5.04\times 10^{1}$	$4.95\times 10^{1}$	**4.80 × 10^1^**
	AvgLatency	3.78	3.04	2.96	3.28	2.97	2.84	**2.82**
	LoadBalance	$5.58\times 10^{1}$	$4.26\times 10^{1}$	$4.33\times 10^{1}$	$4.20\times 10^{1}$	$3.41\times 10^{1}$	$3.38\times 10^{1}$	**3.28 × 10^1^**
	Runtime	**6.50 × 10^−1^**	$6.54\times 10^{1}$	$8.21\times 10^{1}$	$7.52\times 10^{1}$	4.05	4.15	$7.08\times 10^{1}$
	ConstraintViol.	$8.50\times 10^{1}$	$2.30\times 10^{1}$	6.00	7.00	**0.00**	1.00	**0.00**
LS5	Mean Fitness	$1.25\times 10^{7}$	$1.46\times 10^{7}$	$1.44\times 10^{7}$	$1.44\times 10^{7}$	$1.22\times 10^{7}$	$1.20\times 10^{7}$	**1.18 × 10^7^**
	Makespan	$8.05\times 10^{1}$	$9.09\times 10^{1}$	$9.69\times 10^{1}$	$9.31\times 10^{1}$	$7.75\times 10^{1}$	$7.67\times 10^{1}$	**7.45 × 10^1^**
	AvgLatency	5.46	4.60	4.32	4.60	4.23	4.40	**4.17**
	LoadBalance	$8.00\times 10^{1}$	$6.40\times 10^{1}$	$6.55\times 10^{1}$	$6.31\times 10^{1}$	$5.04\times 10^{1}$	$5.00\times 10^{1}$	**4.85 × 10^1^**
	Runtime	**1.05**	$1.09\times 10^{2}$	$1.35\times 10^{2}$	$1.25\times 10^{2}$	6.70	6.85	$1.18\times 10^{2}$
	ConstraintViol.	$1.27\times 10^{2}$	$2.80\times 10^{1}$	7.00	$1.70\times 10^{1}$	**0.00**	1.00	**0.00**

Note: Bold values indicate the best performance among all compared algorithms.

## Data Availability

Data are contained within the article.
